# A comparison of honeybee and scorpion venoms as anticancer agents against three different cancer cell lines: lung, colon, and breast cancer

**DOI:** 10.3389/ftox.2026.1756933

**Published:** 2026-02-25

**Authors:** Fatma H. Galal, Fahad M. Alshammari, Abdulrahman S. Aldaghmi, Elsayed E. Hafez, Ghada M. El-Sayed, Riyadh H. Aeban, Saad A. Alharbi

**Affiliations:** 1 Department of Biology, College of Science, Jouf University, Sakaka, Aljouf, Saudi Arabia; 2 Plant Protection and Biomolecular Diagnosis Department, ALCRI, City of Scientific Research and Technological Applications, Alexandria, Egypt; 3 Microbial Genetic Department, Biotechnology Research Institute, National Research Centre, Cairo, Egypt; 4 Department of Medical Molecular Laboratory, Security Forces Hospital Makkah, Makkah, Saudi Arabia

**Keywords:** bee venom, scorpion venom, GC-MS, gene expression, anticancer markers, molecular docking

## Abstract

Owing to the drawbacks and adverse effects associated with conventional cancer therapies, there is growing interest in identifying effective natural alternatives. In this study, the anticancer potential of honeybee and scorpion venoms was evaluated using three human cancer cell lines: lung adenocarcinoma (A549), colon carcinoma (HCT-116), and breast adenocarcinoma (MDA-MB-231). The chemical composition, biological activity, and molecular interactions of both venoms with key cancer-related targets were investigated through gas chromatography–mass spectrometry (GC-MS), cytotoxicity assays, gene expression analysis, and molecular docking. GC-MS analysis revealed that scorpion venom was predominantly composed of methyl isocyanide, 3-butyn-1-ol, and allene, whereas honeybee venom was characterized by caprylic anhydride, 1,3,5-triazine derivatives, and palmitin as major bioactive constituents. Functional analyses demonstrated that both venoms modulated the expression of genes associated with apoptosis and other cancer-related pathways rather than inducing apoptosis directly. Notably, scorpion venom significantly downregulated the anti-apoptotic gene *Bcl-2*, whereas honeybee venom upregulated its expression, indicating distinct mechanisms of action. Scorpion venom exerted pronounced pro-apoptotic effects, while honeybee venom appeared to act primarily through immunomodulatory and anti-angiogenic pathways. Molecular docking analyses confirmed favorable interactions between venom-derived compounds and key molecular targets, including Bcl-2, Bax, p53, and VEGF, supporting their potential as multi-target anticancer agents. Collectively, these findings demonstrate that honeybee and scorpion venoms possess promising anticancer properties via distinct yet complementary mechanisms, with particular efficacy against lung and breast adenocarcinoma cell lines. The results highlight the potential of these venoms as natural candidates for the development of alternative anticancer therapeutics.

## 1 Introduction

Cancer remains a formidable global health challenge, accounting for nearly 10 million deaths annually (Lin et al., 2021). Lung, breast, and colorectal malignancies rank among the most prevalent, collectively representing over one-third of cancer-related mortality worldwide (El-Fawal et al., 2024). Conventional treatments, including chemotherapy and radiotherapy, frequently suffer from limited selectivity, severe systemic toxicity, and acquired resistance (Cheville et al., 2021). These shortcomings have intensified the search for novel therapeutic agents derived from natural sources, which offer enhanced biocompatibility and multimodal mechanisms of action (Naeem et al., 2022). Because of their ability to selectively target cancer cells while reducing systemic toxicity, natural poisons have emerged as attractive alternatives or complementary strategies to conventional chemotherapy. Many natural toxins, including peptides derived from arthropods, marine organisms, and microorganisms, exert anticancer effects through highly specific molecular mechanisms—such as ion channel modulation, membrane disruption, induction of apoptosis, and inhibition of metastatic pathways—unlike classical chemotherapeutic agents, which often cause severe side effects due to non-specific damage to rapidly dividing healthy tissues (Monti et al., 2023). Advances in peptide engineering and nano-delivery technologies have substantially improved the stability, bioavailability, and tumor selectivity of natural toxins, enabling them to overcome key limitations associated with chemotherapy resistance and drug efflux mechanisms (Dai et al., 2022; Li et al., 2024). Natural toxin–based therapies, including melittin-loaded nanoparticles, chlorotoxin conjugates, and spider-derived cytolytic peptides, have demonstrated strong efficacy against tumors resistant to conventional chemotherapeutic agents (de Melo Carvalho Rocha et al., 2023). Furthermore, natural toxins represent promising candidates for next-generation anticancer therapeutic pipelines, as they provide multimodal effects that simultaneously disrupt cancer cell membranes, modulate signaling pathways, and sensitize tumors to existing treatments (Ghodeif et al., 2025). Consequently, natural toxins are increasingly viewed not merely as adjuncts but as potential alternatives to specific chemotherapy regimens, particularly in cancers where toxicity and resistance limit the effectiveness of traditional therapies.

Arthropod venoms represent particularly promising candidates in this context. For millennia, honeybee (*Apis mellifera*) venom (apitoxin) has been used in traditional medicine across ancient Egyptian, Greek, and Chinese civilizations to treat inflammation, arthritis, and tumors (Kolayli and Keskin, 2020; Rady et al., 2017). Its complex biochemical matrix, comprising melittin, phospholipase A_2_, and apamin, exerts pro-apoptotic, anti-angiogenic, and immunomodulatory effects that have been validated in diverse carcinoma models (Ullah et al., 2023). Similarly, scorpion venoms contain neuroactive peptides (e.g., chlorotoxin) that selectively disrupt ion channels on cancer cells, thereby inhibiting metastasis and triggering mitochondrial apoptosis (Wang et al., 2024; Mendes et al., 2022). Despite these advances, critical knowledge gaps persist. Few studies have conducted comparative analyses of these venoms against multiple human carcinomas, and their molecular interactions with key oncogenic pathways remain incompletely characterized.

Insect venoms remain among the most extensively studied arthropod-derived anticancer agents (Ghodeif et al. 2025). Melittin, the principal component of honeybee venom, has demonstrated broad-spectrum cytotoxic activity through mechanisms involving caspase-dependent apoptosis, reactive oxygen species (ROS) generation, and mitochondrial membrane depolarization (Rady et al., 2017). Recent studies have confirmed its efficacy in glioblastoma, breast, and liver cancer models, particularly when delivered via nanocarrier systems that reduce hemolytic toxicity (Dai et al., 2022; Park et al., 2024; Kong et al., 2021).

In addition to melittin, mastoparan peptides derived from wasp venom have attracted considerable interest due to their ability to induce mitochondrial pore formation and activate G-protein–mediated signaling pathways. Optimized mastoparan analogues have exhibited reduced hemolytic activity alongside enhanced selectivity toward cancer cells (Torres-Duran et al., 2021). Furthermore, ant-derived peptides, such as ponericins and dinoponeratoxins, possess α-helical structures that selectively disrupt cancer cell membranes and have demonstrated cytotoxic activity against leukemia and colon cancer cell lines (Santos et al., 2020; Ferreira et al., 2021). Another important class of arthropod-derived anticancer agents is represented by the proteins found in scorpion venom. The most well-known example is chlorotoxin, a 36–amino acid peptide that interacts with matrix metalloproteinase-2 and chloride channels, conferring remarkable glioma selectivity. Chlorotoxin conjugates have been shown to enhance blood–brain barrier penetration, tumor imaging, and therapeutic targeting (Cao et al., 2022; Gopalakrishnan et al., 2025). In breast and lung cancer models, other scorpion peptides, including BmK AGAP and Rhopalurus toxins, have demonstrated the ability to inhibit metastasis, block angiogenesis, and induce apoptosis (Zhang et al., 2020; Pimentel et al., 2022).

Molecular docking has emerged as a crucial computational tool in modern cancer research, enabling accurate prediction of interactions between anticancer agents and their molecular targets while accelerating drug discovery and reducing experimental time and costs. This approach facilitates the identification of compounds capable of modulating key oncogenic proteins, such as kinases, apoptotic regulators, and DNA repair enzymes, by simulating ligand–receptor binding at atomic resolution (Arjmand et al., 2022). Docking is particularly valuable for natural-toxin-based therapies, such as arthropod venom peptides, as it elucidates how these molecules interact with membrane receptors, ion channels, and proteases that are overexpressed in tumor cells (Almeida et al., 2025; Zhou et al., 2023). Additionally, molecular docking guides the optimization of structure–activity relationships by predicting how chemical modifications may enhance target affinity, reduce toxicity, or increase specificity (Mozaffari et al., 2025). When combined with molecular dynamics simulations and machine-learning scoring functions, docking pipelines enable the screening of thousands of potential anticancer compounds and prioritize the most promising candidates for subsequent *in vitro* and *in vivo* validation (Li et al., 2024). Consequently, molecular docking has become an essential tool in the development of targeted therapies, rational drug design, and personalized oncology (Wang et al., 2025).

By applying an integrated pharmacological and transcriptomic approach, our study addresses these critical knowledge gaps regarding Egyptian honeybee and scorpion venoms. We evaluated their antiproliferative activity in key adenocarcinoma models, including lung (A549), colon (HCT-116), and breast (MDA-MB-231) cancer cell lines, and compared their cytotoxicity against normal human lung fibroblasts (WI-38). Furthermore, the study examined venom-induced transcriptional changes in key molecular pathways by assessing immunomodulatory cytokines (IL-2, IL-6, IL-12), tumor-suppressor signaling (p53), apoptotic regulators (Bcl-2 and Bax), and angiogenic mediators (VEGF). Gas Chromatography–Mass Spectrometry (GC-MS) was employed to characterize the bioactive components of both venoms, providing a comprehensive molecular basis for understanding their distinct anticancer mechanisms. Also, by comparing multiple docking scenarios, we seek to clarify how these molecules exert their biological activity and to identify the most favorable binding modes for therapeutic applications.

## 2 Materials and methods

### 2.1 Honeybee and scorpion venom acquisition and preparation

Lyophilized venoms from honeybees (*A. mellifera*) and scorpions (*Leiurus quinquestriatus*) were sourced from a private Egyptian farm (Ahmed Ragab Dakroney, Albaheria governorate, Egypt). The venom powders were transported on dry ice to preserve stability and stored at −20 °C until use. Immediately prior to experimentation, working solutions were prepared in sterile phosphate-buffered saline (PBS: pH 7.4) to prevent bioactive degradation.

### 2.2 Cell culture maintenance

Four human cell lines were utilized: WI-38 normal lung fibroblasts (normal control), A549 lung adenocarcinoma, HCT-116 colon carcinoma, and MDA-MB-231 breast adeno-carcinoma cells. All lines were procured from Catalent Biologics (Anagni, Italy) and maintained in Dulbecco’s Modified Eagle Medium (DMEM) supplemented with 10% fetal bovine serum (FBS) and 1% penicillin-streptomycin antibiotic mixture (100 U/mL penicillin, 100 μg/mL streptomycin). Cells were incubated at 37 °C under 5% CO_2_ with 95% humidity, with media refreshed every 48 h.

### 2.3 Study of hemolytic activity

Hemolysis of human red blood cells (RBCs) was assessed following the method of Yousefpoor et al. (2019) to determine the concentration of venom causing 50% complement hemolytic activity (CH_50_). Fresh human blood was collected in heparinized tubes (20 IU per 5 mL) and incubated at 37 °C for 10 min. Samples were then centrifuged at 3,000 rpm for 10 min to remove plasma. The RBC pellet was washed five times with phosphate-buffered saline (PBS) until the supernatant became clear, and the cells were finally resuspended in PBS to a 2% RBC suspension. Serial dilutions of each venom were incubated with the RBC suspension at 37 °C for 2 h. Triton X-100 served as the positive control, and PBS served as the negative control. After incubation, tubes were centrifuged at 3,500 rpm for 10 min, and absorbance of the supernatant was measured at 540 nm using PBS as the blank. All experiments were performed in triplicate. Hemolysis percentage was calculated using Equation 1 (Yousefpoor et al., 2019).

$$\text{Hemolysis }\left(\%\right)=\frac{\text{Sample}\;\text{absorbance}-\text{Negative}\;\text{control}\;\text{absorbance}}{\text{Positive}\;\text{control}\;\text{absorbance}-\text{Negative}\;\text{control}\;\text{absorbance}}\times 100$$ (1)

### 2.4 Cytotoxicity and selectivity assessment of bee venom (BV) and scorpion venom (SV)

Cytotoxicity was assessed using the MTT colorimetric assay. Cells, WI-38 normal lung fibroblasts, A549 lung adenocarcinoma, HCT-116 colon carcinoma, and MDA-MB-231 breast adeno-carcinoma were seeded in 96-well plates in triplicate at a density of 1 × 10^4^ cells per well and allowed to adhere for 24 h. Stock solutions of honeybee and scorpion venoms (1 mg/1 mL) were prepared, from which a series of dilutions was generated (0.1, 0.19, 0.39, 0.78, 1.56, 3.1, 6.25, 12.5, 25, and 50% w/v (µg/100 µL). Cells were then treated with the respective venom dilutions for 48 h. Following treatment, 20 μL of MTT solution (5 mg/mL in PBS) was added to each well and incubated for 4 h at 37 °C. The resulting formazan crystals were dissolved in 200 μL dimethyl sulfoxide (DMSO) per well. Absorbance was measured at 570 nm using a BioTek Synergy HT microplate reader. Cell viability was calculated according to Equation 2, and IC_50_ values were determined from dose–response curves using nonlinear regression analysis in GraphPad Prism 9.0 (GraphPad Software, San Diego, CA, USA).

$$\%\text{ Cell viability}=\frac{\text{OD}\;\text{Sample}}{\text{OD}\;\text{Control}}\times 100$$ (2)

To estimate the therapeutic window of each venom, the selectivity index (SI) was calculated as follows:

$$\text{SI}=\frac{{\text{IC}}_{50}\;\text{of}\;\text{normal}\;\text{cells}}{{\text{IC}}_{50}\;\text{of}\;\text{cancer}\;\text{cells}}$$

According to Valdés-García et al. (2003), compounds with an SI > 1 are considered selective toward cancer cells, indicating potential therapeutic relevance.

### 2.5 Gene expression profiling

It conducted on A549 lung adenocarcinoma cells. This choice was guided by the parallel use of WI-38 normal lung fibroblasts as the corresponding non-tumor control. A549 cells (1 × 10^4^ cells per well) were seeded into 96-well plates in triplicates and incubated for 24 h. The cells were then treated with IC_50_ concentrations of the venoms in fresh culture medium, with each treatment performed in triplicate wells, and incubated for an additional 48 h. Untreated wells served as negative controls. Following the incubation period, cells were harvested for RNA isolation. Total RNA was isolated using the RNeasy Mini Kit (Qiagen) following the manufacturer’s protocol. RNA purity and concentration were verified spectrophotometrically (Nanodrop 2000), with A_260_/A_280_ ratios >1.8 considered acceptable. cDNA was synthesized from 500 ng RNA using RevertAid Reverse Transcriptase (Thermo Scientific) primed with oligo (dT) under thermal conditions of 42 °C for 60 min followed by 95 °C for 5 min. Quantitative PCR reactions (25 μL total volume) contained 12.5 μL SYBR Green Master Mix (Fermentas), 1 μL cDNA template (50 ng), 1 μL each of forward and reverse primers (10 pmol/μL; sequences provided in Table 1), and 9.5 μL nuclease-free water. Amplification was performed on a Rotor-Gene 6000 system using the following protocol: initial denaturation at 95 °C for 10 min; 40 cycles of denaturation (95 °C, 15 s), annealing (60 °C, 30 s), and extension (72 °C, 30 s); followed by melt curve analysis from 65 °C to 95 °C in 0.5 °C increments. *GAPDH* served as the endogenous reference gene, and relative expression of target genes (*IL-2*, *IL-6*, *IL-12*, *P53*, *Bcl-2*, *Bax* and *VEGFR*) was calculated using the 2^(−ΔΔCT)^ method.

**TABLE 1:** The oligonucleotide sequences of the primers used in qRT-PCR study.

Primer	Direction	Sequences 5′-3′
P-53	F	TAA​CAG​TTC​CTG​CAT​GGG​CGG​C
	R	AGG​ACA​GGC​ACA​AAC​ACG​CAC​C
Bcl-2	F	TTG​TGG​CCT​TCT​TTG​AGT​TCG​GTG
	R	GGT​GCC​GGT​TCA​GGT​ACT​CAG​TCA
IL-2	F	AAG​AGT​CAT​CAG​AAG​AGG​AAA
	R	AACCTT GGGCATGTAGAAGT
IL-6	F	CCA​GGA​TCC​CAG​CTA​TGA​ACT​CCC​TCT​TC
	R	GGA​GAA​TTC​GCT​ACT​TCA​TCC​GAA​TGA​CTC
IL-12	F	CAC​CAC​CTG​CCC​CAC​CTC​AG
	R	CTA​CGA​AGA​ACT​CAG​ATA​G
VEGFR	F	GAG​ATG​AGC​TTC​CTA​CAG​CAC
	R	TCA​CCG​CCT​CGG​CTT​GTC​ACA​T
Bax	F	TGG​CAG​CTG​ACA​TGT​TTT​CTG​AC
	R	TCA​CCC​AAC​CAC​CCT​GGT​CTT
GAPDH	F	GTC​TCC​TCT​GAC​TTC​AAC​AGC​G
	R	ACC​ACC​CTG​TTG​CTG​TAG​CCA​A

### 2.6 Bioactive compounds identification by gas chromatography–mass spectrometry (GC-Mass)

Bioactive constituents were characterized using gas chromatography-mass spectrometry (GC-MS). Venom samples were analyzed on a Trace GC Ultra-ISQ spectrometer (Thermo Scientific) equipped with a DB-5MS capillary column (30 m × 0.25 mm, 0.25 μm film thickness). The oven temperature program was 50 °C (2 min), ramped to 300 °C at 10 °C/min, held for 10 min. Helium served as the carrier gas (1.0 mL/min flow rate). Mass spectra were acquired in electron ionization mode (70 eV) over 40–600 m/z. Compounds were identified by matching retention times and mass fragmentation patterns against Wiley 11 and NIST 2020 libraries.

### 2.7 Prediction of drug-likeness for compounds in honeybees and scorpion venoms

The drug-likeness of bioactive compounds from honeybee and scorpion venoms was evaluated using Lipinski’s Rule of Five (Lipinski et al., 2001; Lipinski, 2004). This rule predicts the likelihood that a compound will exhibit favorable pharmacokinetic properties when administered orally. According to Lipinski’s criteria, a compound is considered a potential drug candidate if it satisfies the following conditions: calculated logP (ClogP) ≤ 5, molecular weight (MW) ≤ 500 g/mol, ≤ 10 hydrogen bond acceptors, and ≤ 5 hydrogen bond donors (de Brito, 2011). To further assess the pharmacological potential, a drug score was calculated for each compound, where higher values indicate greater drug-likeness and better suitability as a therapeutic candidate (Oduselu et al., 2019). The Swiss ADME web tool (Daina et al., 2017) was used to predict and analyze physicochemical properties, including the number of rotatable bonds, hydrogen bond donors and acceptors, lipophilicity (logP), and molecular weight. Compounds that complied fully with Lipinski’s Rule of Five were selected for subsequent molecular docking studies, ensuring their compatibility with oral bioavailability requirements.

### 2.8 Computational studies

#### 2.8.1 Selection of protein models, prediction of active sites and preparation of ligands

The three-dimensional (3D) structures of the target protein receptors were retrieved from the Research Collaboratory for Structural Bioinformatics Protein Data Bank (RCSB-PDB). The selected proteins and their corresponding PDB identification codes are listed in Table 2. For *3DCY, 6WDP, 4S0O,* and *4O9H*, the active sites of these proteins were predicted using the COACH server (Yang et al., 2013), which integrates multiple algorithms to identify potential ligand-binding residues based on structural and sequence features. For the proteins with PDB IDs 1M48 (interleukin-2) and 6O0K (B-cell lymphoma 2; BCL-2), 6GQO (Vascular Endothelial Growth Factor Receptor 2 - VEGFR-2). The co-crystallized ligands—FRG for IL-2, venetoclax for BCL-2, and F82 for VEGFR-2 —were used to define the binding sites for molecular docking studies. Prior to docking, all protein structures were refined and prepared using *BIOVIA Discovery Studio 2021 Client* (Dassault Systèmes). This process involved the removal of non-essential molecules, including water, side chains, heteroatoms (HETATM), and native ligands, followed by energy minimization to stabilize the structures. The tertiary structures of the selected proteins were visualized and analyzed using PyMOL version 2.5.0 (Schrödinger, LLC), which facilitated confirmation of structural integrity and active-site orientation prior to subsequent docking analysis. The compounds detected by GC-MS analysis of bee venom and scorpion venom were prepared for docking as follow: the two-dimensional (2D) structures were obtained from the PubChem database in SDF format. The ligand structures were first converted to MOL2 format using Open Babel (O'Boyle et al., 2011) and subsequently converted to PDBQT format using AutoDock Tools for compatibility with docking simulations. Hydrogen atoms were added to the three-dimensional (3D) structure of the target protein using the MGL Tools package of AutoDock Vina (Eberhardt et al., 2021).

**TABLE 2:** PDB IDs of the receptor proteins used in the study.

Receptors	PDB IDs	References
Tp53	3DCY	Rath et al. (2021)
Il2	1M48	Kalsoom et al. (2013)
IL12	6WDP	Glassman et al. (2021)
Bax	4S0O	Gitego et al. (2021)
BCL2	6O0K	Tondar et al. (2024)
IL6	4O9H	Klarenbeek et al. (2016)
VEGFR-2	6GQO	Ramzan et al. (2023)

#### 2.8.2 Molecular docking analysis

Molecular docking simulations were performed using AutoDock Tools version 1.5.6 and AutoDock Vina v1.0 to predict the binding interactions between selected ligands and their respective receptor proteins. Prior to docking, all receptor proteins were preprocessed in AutoDock Tools 1.5.6 by incorporating polar hydrogen atoms and assigning Gasteiger partial charges. The prepared protein structures were then saved in PDBQT format for docking analysis. The active sites of each protein, as predicted by the COACH server, were used to define the grid box dimensions and center coordinates for the docking process. During the simulation, The number of genetic algorithm runs was set to 30, and all other parameters were maintained at their default values. Docking results were evaluated using the AutoDock scoring function, and the protein–ligand complexes were ranked based on their binding affinities (ΔG, kcal/mol). The lowest binding energy (most negative value) was considered indicative of the most stable and favorable interaction. Post-docking visualization and interaction analysis were performed using BIOVIA Discovery Studio 2021 Client (Dassault Systèmes, San Diego, CA, USA) and PyMOL version 2.5.0. For each ligand, ten docking poses were generated, and the conformation showing the strongest binding affinity was selected for detailed investigation.

### 2.9 Statistical analysis

All experiments were performed in triplicate (n = 3). Data are presented as mean ± standard deviation (SD). Statistical significance was determined using one-way ANOVA followed by Fisher’s LSD *post hoc* test (*p* ≤ 0.05) in Costat software (Cohort Analytics). Dose-response curves were fitted using four-parameter logistic regression in GraphPad Prism 9.0.

## 3 Results

### 3.1 Cytotoxicity on normal human lung fibroblasts

Scorpion and honeybee venoms exhibited concentration-dependent cytotoxic effects on WI-38 normal lung fibroblast cells. Bee venom was significantly less toxic than scorpion venom, with IC_50_ values of 9.63% and 5.02%, respectively (p < 0.05). At the highest tested concentration (50%), bee venom inhibited cell viability by 76%, whereas scorpion venom caused 96% inhibition, demonstrating the markedly higher cytotoxicity of scorpion venom (p < 0.01).

### 3.2 Study of hemolytic activity of BV and SV

**FIGURE 1:**
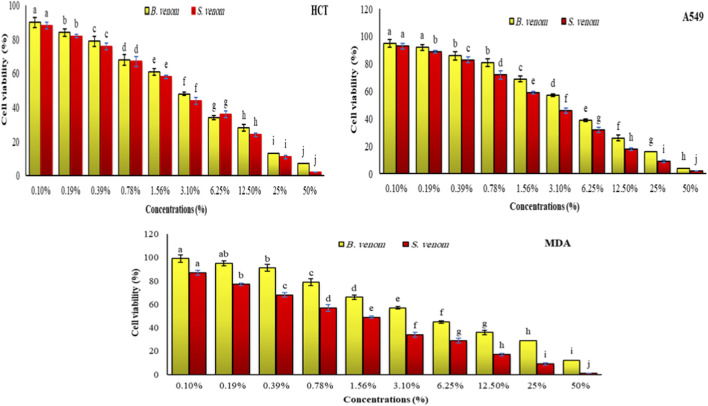
The effect of different concentrations (0.1, 0.19, 0.39, 0.78, 1.56, 3.1, 6.25, 12.5, 25% and 50%) of B. venom and S. venom on hemolysis of human RBCs. Data are means of three different replicates ± standard deviations (SD). Different letters on each row illustrate statistically significant variations at P ≤ 0.05.

As shown in Figure 1, the hemolysis assay indicates that scorpion venom (SV) is more potent than bee venom (BV), as evidenced by its lower CH_50_ value (∼4% for SV compared with ∼10% for BV). This result demonstrates that SV induces 50% red blood cell lysis at a substantially lower concentration than BV.

### 3.3 Anticancer activity and cell availability on three different carcinoma cell lines

**FIGURE 2:**
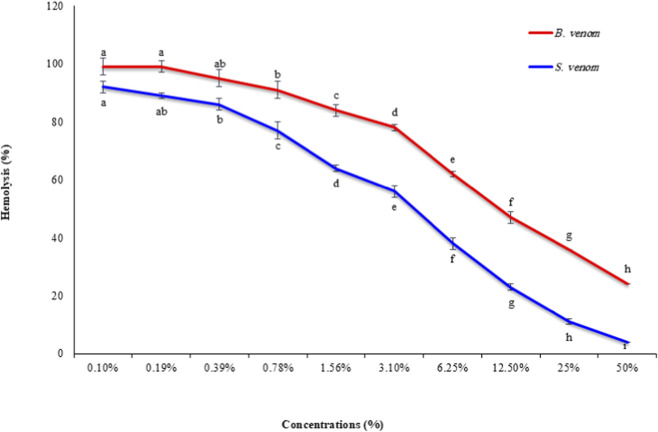
The effect of different concentrations (0.1, 0.19, 0.39, 0.78, 1.56, 3.1, 6.25, 12.5, 25% and 50%) of B. venom and S. venom on the viability of lung (A549), colon (HCT) and breast (MDA) cell lines. Data are means of three different replicates ± standard deviations SD. Different letters on column illustrate statistically significant variations at *p* ≤ 0.05.

From Figure 2, Concentration–response analyses demonstrated strong anticancer activity of both scorpion and honeybee venoms across all tested cancer cell lines, although their cytotoxic effects varied in a cell line–dependent manner. In lung cancer cells (A549), scorpion venom was more potent than honeybee venom, with IC_50_ values of 3.2% and 5.3%, respectively. At the highest tested concentration (50%), cell viability was reduced by 98% with scorpion venom and 96% with honeybee venom, indicating near-complete inhibition of A549 cell proliferation. In colon cancer cells (HCT-116), both venoms exhibited comparable efficacy, each with an IC_50_ of 4.3%. Maximum inhibition at 50% concentration was slightly higher for scorpion venom (93%) than for honeybee venom, reflecting similar cytotoxic potential in this cell line. In contrast, breast cancer cells (MDA-MB-231) showed pronounced sensitivity to scorpion venom, which exhibited an IC_50_ of 1.1% and achieved 99% inhibition at 50% concentration, making it the most effective treatment among those tested. Honeybee venom displayed moderate activity against this cell line, with an IC_50_ of 5.7% and 88% inhibition, highlighting differential venom sensitivity in breast cancer cells. Overall, these results indicate that while both venoms possess strong anticancer activity against lung and colon cancer cells, scorpion venom consistently demonstrates greater cytotoxic efficacy than honeybee venom, particularly against aggressive breast adenocarcinoma. These findings suggest the presence of venom-specific cytotoxic mechanisms with potential application in targeted anticancer therapies.

### 3.4 Assessment of selectivity index

The selectivity index (SI) was calculated to evaluate the therapeutic window of bee and scorpion venoms across different cancer cell lines (Table 3). Both venoms exhibited SI values greater than 1 in all tested cancer models, indicating preferential cytotoxicity toward malignant cells relative to normal fibroblasts. In lung cancer cells (A549), both venoms showed moderate selectivity, with SI values of 1.82 for bee venom and 1.56 for scorpion venom. In colon cancer cells (HCT-116), bee venom demonstrated higher selectivity (SI = 2.24) than scorpion venom (SI = 1.17), suggesting a more favorable therapeutic profile in this cell line. Noticeably, breast cancer cells (MDA-MB-231) exhibited pronounced differential selectivity between the two venoms. Scorpion venom displayed a markedly high SI value (4.56), reflecting strong preferential toxicity toward aggressive breast cancer cells and a broad therapeutic window, whereas bee venom showed only moderate selectivity (SI = 1.69). Overall, these findings highlight venom-specific differences in selectivity, with scorpion venom showing superior tumor selectivity against breast adenocarcinoma cells, while bee venom demonstrates comparatively better selectivity in colon cancer cells.

**TABLE 3:** Cytotoxic activity and selectivity index (SI) of bee and scorpion venoms against normal and cancer cell line.

Cell type	Venoms			
	Bee venom		Scorpion venom	
	IC50	SI	IC50	SI
Normal cell	9.63	--	5.02	--
A549	5.3	1.82	3.2	1.56
HCT	4.3	2.24	4.3	1.17
MDA	5.7	1.69	1.1	4.56

### 3.5 Cancer marker genes and gene expression in the examined cell lines

**FIGURE 3:**
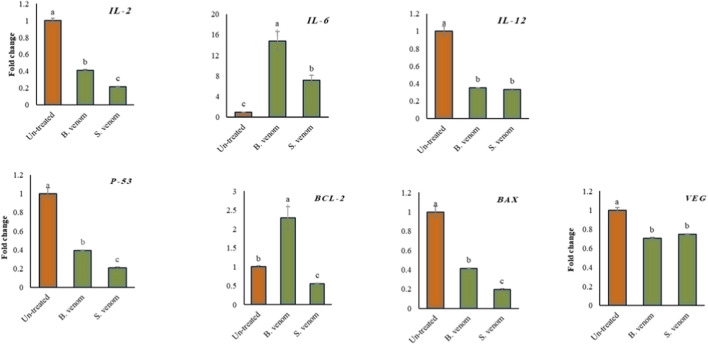
The quantitative relative expression level of (*IL-2, IL-6, IL-12, P-53, B-cl2, Bax and VEG)*, genes of lung cancer cell lines un-treated compared to bee and scorpion venoms treated. Data are means of three different replicates ± standard deviations (SD). Different letters on column illustrate statistically significant variations at *p* ≤ 0.05.

As illustrated in Figure 3, qquantitative PCR analysis of A549 lung adenocarcinoma cells treated with IC_50_ concentrations of honeybee and scorpion venoms reveal notable, gene-specific changes in cancer-associated pathways. Immune-related genes were differentially regulated: IL-6 was significantly upregulated, increasing 14.8-fold with honeybee venom and 7.2-fold with scorpion venom, whereas IL-2 and IL-12 were consistently downregulated by both venoms (p < 0.05), indicating selective inhibition of certain immune signaling pathways. Apoptosis-associated genes also showed divergent responses: the anti-apoptotic gene Bcl-2 was suppressed by scorpion venom but upregulated 2.3-fold by honeybee venom, while the pro-apoptotic gene Bax was downregulated under both treatments, suggesting modulation of intrinsic apoptotic pathways. In terms of angiogenesis and tumor suppression, both venoms caused significant downregulation of P53 (p < 0.01), and honeybee venom exerted a more pronounced effect on VEGFR-2, a key mediator of angiogenesis, than scorpion venom. Overall, these results indicate that venom treatments differentially modulate immune, apoptotic, and angiogenic genes, with honeybee venom predominantly influencing immune and angiogenic pathways, whereas scorpion venom primarily targets anti-apoptotic factors. This venom-specific gene expression profile likely contributes to the observed differences in cytotoxicity and selectivity in cancer cells.

### 3.6 GC-mass analysis and bioactive compound profiling

**FIGURE 4:**
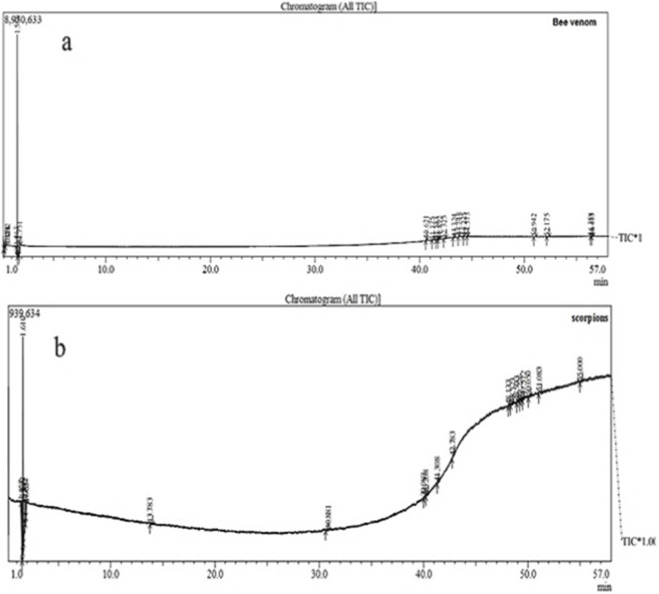
GC-MS chromatogram of Bee venom **(a)** and Scorpion venom **(b)**.

Gas chromatography (GC) analyses (Tables 4 and 5; Figure 4) reveal that both honeybee and scorpion venoms contain complex mixtures of low-molecular-weight compounds, but with distinct chemical profiles and relative abundances. Honeybee venom is dominated by ethane, 1-chloro-1-fluoro (42.79%) and acetonitrile (24.97%), with smaller amounts of furazan (3.32%), caprylic anhydride (2.41%), and heterocyclic derivatives such as 1,3,5-triazine. Most of these compounds have molecular weights between 40 and 330 g/mol and are small, polar, or halogenated molecules capable of interacting with diverse biological targets. In contrast, scorpion venom contains higher proportions of methyl isocyanide (28.93%), 3-butyn-1-ol (20.74%), and allene (∼25% cumulatively), while N-ethyl-N′-nitroguanidine (4.60%) and cis-aconitic anhydride (∼10%) are present in smaller amounts. The compounds in scorpion venom are generally reactive small molecules with molecular weights ranging from 40 to 179 g/mol, including isocyanides, alkynols, nitroguanidines, and dioxolanes, suggesting strong potential for bioactive interactions. Regarding abundance, scorpion venom exhibits a more uniform chemical distribution, with the top three compounds comprising approximately 75% of the total area. By contrast, honeybee venom is dominated by a few major compounds, with ethane, 1-chloro-1-fluoro alone representing over 40% of the total. Chemical diversity also differed: scorpion venom contains structurally diverse reactive compounds, such as isocyanides and alkynols, which may enhance cytotoxic and apoptotic effects. Although honeybee venom is less structurally diverse, it contains heterocyclic and halogenated molecules, including caprylic anhydride and triazine, which may confer drug-like properties based on Lipinski analysis. The high concentration of a few key compounds in honeybee venom may influence bioavailability, whereas scorpion venom’s broader chemical diversity likely contributes to multi-target cytotoxicity and apoptotic induction. Collectively, these observations suggest that honeybee venom may be better suited for general immunomodulatory or angiogenesis-targeting applications, whereas scorpion venom exhibits greater selective anticancer efficacy.

**TABLE 4:** The most abundant compounds of Bee venom using GC-MS spectral analysis.

R.T.	Area%	Compound	MW	MF	CAS#
0.23	1.49	Propadiene	40	C3H4	74-99-7
0.27	1.86	2-Hexynoic acid	112	C6H8O2	764-33-0
0.43	3.32	Furazan	70	C2H2N2O	3984-19-8
0.55	0.87	2-Methyl-1,5-(4H)-dihydropyrido-(2,3-b)1,4-diazepine-4-one	175	C9H9N3O	17260-05-8
1.49	42.79	Ethane, 1-chloro-1-fluoro	82	C2H4ClF	1615-75-4
1.60	24.97	Acetonitrile	41	C2H3N	75-05-8
1.82	4.26	2-Butanone	72	C4H8O	78-93-3
1.90	1.10	Butanedioic acid	118	C4H6O4	97-65-4
2.08	1.80	Isobutyric acid, allyl ester	128	C7H12O2	15727-77-2
2.24	1.76	2-Pentanone	86	C5H10O	107-87-9
2.38	1.51	cis-Aconitic anhydride	156	C6H4O5	6318-55-4
2.49	1.35	l-Felinine	207	C8H17NO3S	0-00-0
43.17	0.82	Palmitin, 2-mono-	330	C19H38O4	23470-00-0
43.67	0.31	3-Ethyl-3-heptanol	144	C9H20O	19780-41-7
44.71	1.55	2,5-Furandione, dihydro-3-methylene-	112	C5H4O3	2170-03-8
46.17	2.13	1,3,5-Triazine, 2,4,6-tris(cyanomethoxy	246	C9H6N6O3	891-64-5
46.41	0.92	4-Spirohexanone, 5,5-dichloro-	164	C6H6Cl2O	138469-24-6
46.66	1.52	Methanethioamide, N,N-dimethyl-	89	C3H7NS	758-16-7
47.23	0.24	n-Dodecyl methacrylate	254	C16H30O2	142-90-5
50.95	2.41	Caprylic anhydride	270	C16H30O3	623-66-5

**TABLE 5:** The most abundant compounds of Scorpion venom using GC-MS spectral analysis.

R.T.	Area%	Name	MW	MF	CAS#
1.54	14.27	Allene	40	C3H4	463-49-0
1.61	28.93	Methyl isocyanide	41	C2H3N	593-75-9
1.74	20.74	3-Butyn-1-ol	70	C4H6O	927-74-2
1.83	4.60	N-Ethyl-N′-nitroguanidine	132	C3H8N4O2	39197-62-1
1.96	3.48	cis-Aconitic anhydride	156	C6H4O5	6318-55-4
13.78	1.19	Sulphonyl diacetonitrile	144	C4H4N2O2S	0-00-0
39.98	1.46	1,2-Propadiene-1,3-dione	68	C3O2	504-64-3
41.30	1.22	2-Methylaminomethyl-1,3-dioxolane	117	C5H11NO2	57366-77-5
48.52	1.43	N-Methoxymethyl-N-methylformamide	103	C4H9NO2	5129-79-3
48.93	1.84	Ethanamine, 2-(2,6-dimethylphenoxy)-N-methyl-	179	C11H17NO	14573-22-9
49.22	1.18	1,4,7,10-Tetraoxacyclododecane	176	C8H16O4	294-93-9
51.08	1.91	p-Dioxane, methylene-	100	C5H8O2	3984-19-8

### 3.7 Results of (Lipinski’s rule of five analysis)

Lipinski’s Rule of Five was applied to evaluate the drug-likeness of bioactive small molecules in scorpion and honeybee venoms (Supplementary Table S1). All compounds fully complied with Lipinski’s criteria, showing no violations. Honeybee venom compounds had iLogP values ranging from 0.32 to 4.50, indicating a balanced hydrophobic–hydrophilic profile conducive to passive membrane permeability. Molecular weights ranged from 40.06 to 330.50 g/mol, well below the 500 g/mol threshold, and the number of hydrogen bond donors (0–3) and acceptors (0–9) suggested potential for meaningful interactions with target proteins. Scorpion venom compounds exhibited molecular weights of 40.06–179.26 g/mol and iLogP values between 0.00 and 2.68, reflecting their small size, chemical flexibility, and compliance with drug-likeness criteria. Overall, both venoms contain low-molecular-weight, structurally diverse molecules with favorable physicochemical properties, supporting their potential as hit or lead compounds in early drug discovery, with promising oral absorption, permeability, and suitability for molecular docking and ADME/Tox studies.

### 3.8 Molecular docking

Molecular docking analysis was conducted to predict ligand–receptor binding affinity and stability through hydrophobic interactions, hydrogen bonding, and van der Waals forces (Safwat et al., 2021). In total, twenty compounds from honeybee venom and twelve from scorpion venom, identified by GC–MS, were docked against selected cancer-related protein targets using AutoDock. Binding affinity was evaluated based on binding energy values, where lower energies indicate stronger and more stable interactions (Pooja et al., 2025). Binding energies of < −4.25, −5.0, and −7.0 kcal/mol were considered indicative of certain, good, and strong binding, respectively (Merizka et al., 2024). All compounds were further assessed for drug-likeness and ranked according to their binding energies.

Except for IL-12, all target proteins exhibited high binding affinities with comparable compounds from both venoms. Among honeybee venom constituents, Palmitin, 2-mono; Caprylic anhydride; 1,3,5-triazine, 2,4,6-tris(cyanomethoxy); n-dodecyl methacrylate; and cis-aconitic anhydride demonstrated the strongest interactions. Scorpion venom compounds showing notable binding toward multiple targets included N-ethyl-N′-nitroguanidine, Ethanamine, 2-(2,6-dimethylphenoxy)-N-methyl-, and 1,4,7,10-tetraoxacyclododecane (Supplementary Table S2).

**FIGURE 5:**
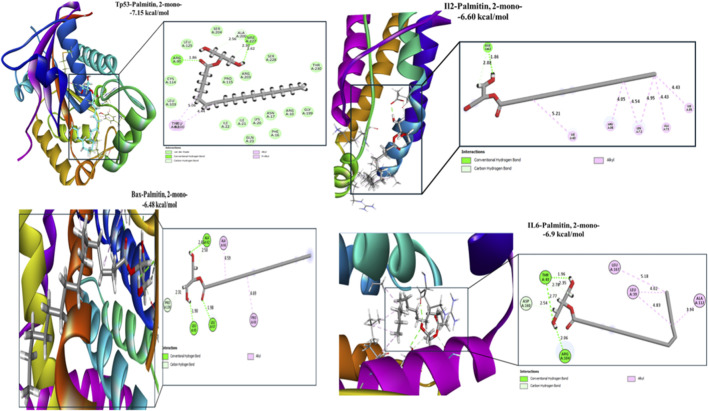
Docking interactions of Palmitin, 2-mono -the most potent bioactive compound derived from bee venom with active-site residues of target protein receptors.

**FIGURE 6:**
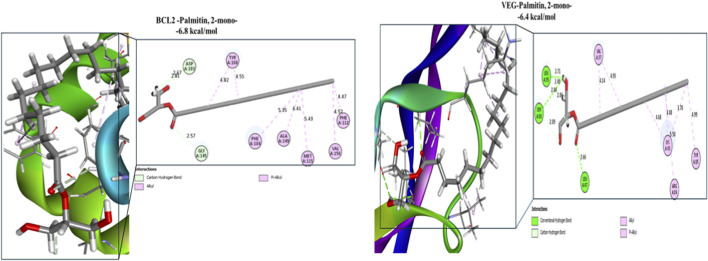
Docking interactions of Palmitin, 2-mono with the active-site residues of BCL2 and VEGFR-2.

Palmitin, 2-mono exhibited the highest overall affinity across all evaluated targets, with binding energies of −7.15, −6.60, −6.48, −6.82, −6.90, and −8.47 kcal/mol for Tp53, IL-2, Bax, BCL-2, IL-6, and VEGFR-2, respectively. These interactions were primarily stabilized by hydrogen bonding and hydrophobic contacts, including hydrogen bonds with ARG90 and MSE227 (Tp53), GLU62 (IL-2), ALA42, LEU45, and LEU47 (Bax), ARG104 and THR43 (IL-6), and LEU35, SER50, and LEU47 (VEGFR-2), indicating strong and stable ligand–receptor complexes (Figures 5, 6).

Caprylic anhydride also showed high binding affinities (−7.9 to −5.8 kcal/mol) with all receptors except IL-12, driven mainly by hydrogen bonding and hydrophobic interactions (Supplementary Figure S1). Similarly, 1,3,5-triazine, 2,4,6-tris(cyanomethoxy) (Supplementary Figure S2) and n-dodecyl methacrylate (Supplementary Figure S3) formed stable complexes with most targets, exhibiting binding energies between −6.45 and −5.15 kcal/mol. Cis-aconitic anhydride, detected in both venoms, interacted favourably with all proteins, including IL-12, through combined hydrogen bonding and hydrophobic forces (Supplementary Figure S4).

**FIGURE 7:**
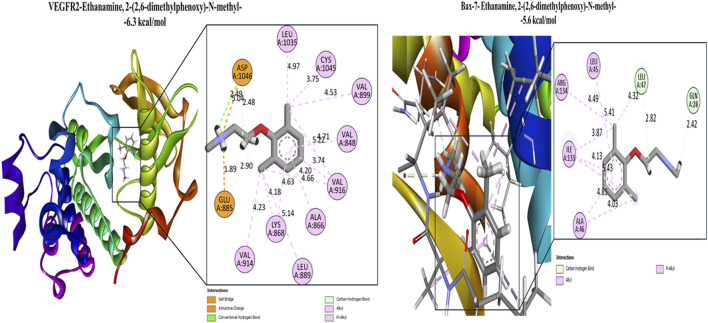
Docking interactions of Ethanamine, 2-(2,6-dimethylphenoxy)-N-methyl the most potent bioactive compound derived from scorpion venom with the active-site residues of VEGFR-2 and IL6.

**FIGURE 8:**
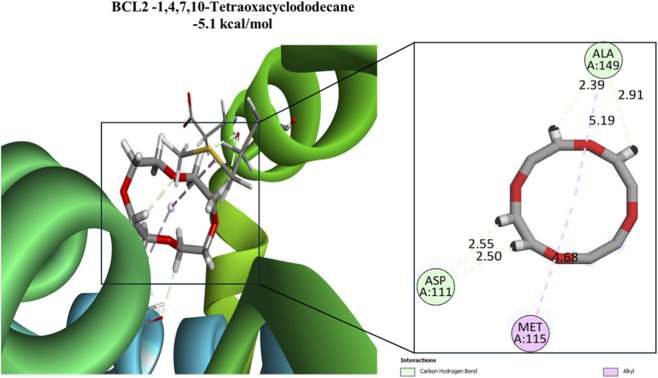
Docking interactions of 1,4,7,10-tetraoxacyclododecane—among the most potent bioactive compound derived from scorpion venom—showing the highest binding affinity toward the active-site residues of BCL-2.

Among scorpion venom compounds, Ethanamine, 2-(2,6-dimethylphenoxy)-N-methyl- showed preferential binding to VEGFR-2, Bax (Figure 7) and IL-6 (Supplementary Figure S5), with energies ranging from −6.3 to −5.2 kcal/mol. N-ethyl-N′-nitroguanidine displayed consistent binding across all targets (−5.3 to −4.3 kcal/mol), stabilized by hydrogen bonding and attractive charge interactions (Supplementary Figure S6). In addition, 1,4,7,10-tetraoxacyclododecane interacted with BCL-2 (Figure 8), VEGFR-2, Bax, and IL-6 (−5.7 to −5.1 kcal/mol), predominantly via hydrophobic interactions (Supplementary Figure S7). N-Methoxymethyl-N-methylformamide also demonstrated strong binding (−5.2 kcal/mol), mediated by hydrophobic and hydrogen bonding interactions (Supplementary Table S2; Supplementary Figure S8).

The strong affinities of Palmitin, 2-mono (−8.47 kcal/mol) and Caprylic anhydride (−7.9 kcal/mol) toward VEGFR-2, Tp53, and IL-2 were consistent with qRT-PCR gene expression results, supporting their role in modulating VEGFR-2, Tp53, and IL-2 expression. At the IL-2 binding site, residues Arg38, Thr41, Phe42, Phe44, Lys43, Tyr45, Glu68, and Leu72 were mainly involved in hydrophobic interactions, while Arg38 and Val69 participated in hydrogen bonding, underscoring the distinct binding behaviour of IL-12.

BCL-2, a key anti-apoptotic regulator overexpressed in several cancers (Sathishkumar et al., 2012), showed strong interactions with Caprylic anhydride (−6.2 kcal/mol), Palmitin, 2-mono (−6.8 kcal/mol), 1,4,7,10-tetraoxacyclododecane (−5.1 kcal/mol), and Ethanamine, 2-(2,6-dimethylphenoxy)-N-methyl- (−4.8 kcal/mol) within the Venetoclax binding region. These interactions were stabilized by hydrophobic contacts with MET115 and PHE112 and hydrogen bonds with ALA100 and ALA149, mirroring the binding pattern of Venetoclax and highlighting the therapeutic potential of these natural compounds. Similar observations have been reported for ginsenosides from Panax ginseng and phytochemicals from Catharanthus roseus (Sathishkumar et al., 2012; Alsaif et al., 2024).

VEGFR-2 docking revealed that Palmitin, 2-mono and Caprylic anhydride exhibited the strongest affinities among honeybee venom compounds, followed by n-dodecyl methacrylate, 1-Felinine (Supplementary Figure S9), and 1,3,5-triazine, 2,4,6-tris(cyanomethoxy). Key interacting residues included CYS919 and ASP1046 (hydrogen bonding) and LEU889, VAL916, and LEU1035 (hydrophobic contacts), consistent with the binding mode of the reference inhibitor sorafenib (−9.7 kcal/mol) (Supplementary Figure S10). Comparable binding trends were observed for scorpion venom compounds, particularly Ethanamine, 2-(2,6-dimethylphenoxy)-N-methyl- and 1,4,7,10-tetraoxacyclododecane.

## 4 Discussion

The cytotoxic profiling of scorpion and honeybee venoms revealed clear differences in both potency and selectivity across normal and malignant cell models. Honeybee venom exhibited a comparatively safer profile toward normal human lung fibroblasts (WI-38), which can be attributed to the physicochemical characteristics of its bioactive constituents. In contrast, scorpion venom contains smaller, highly reactive molecules capable of broad membrane interactions, resulting in stronger but less selective cytotoxicity (Ortiz-Andrade et al., 2020). This differential toxicity supports the concept of a therapeutic window, wherein honeybee venom may offer anticancer efficacy with reduced off-target toxicity, while scorpion venom—despite its superior potency—poses a greater risk to healthy cells (El-Qassas et al., 2024; Ding et al., 2023). Consistent with these findings, both venoms demonstrated marked anticancer activity against lung adenocarcinoma (A549), colon carcinoma (HCT-116), and breast adenocarcinoma (MDA-MB-231) cells. Scorpion venom was consistently more potent, particularly against MDA-MB-231 cells, where it achieved an IC_50_ of 1.1% and near-complete inhibition at higher concentrations. This enhanced activity likely reflects the presence of highly reactive venom-derived molecules capable of simultaneously targeting apoptotic, angiogenic, and inflammatory pathways (Bagyalakshmi et al., 2019; Kim et al., 2025). Both venoms showed comparable IC_50_ values against HCT-116 cells, suggesting shared mechanisms such as oxidative stress induction, mitochondrial dysfunction, and cell-cycle arrest (Małek et al., 2023). The pronounced sensitivity of MDA-MB-231 cells to scorpion venom highlights venom-specific cytotoxic signatures, possibly linked to modulation of VEGF-driven angiogenesis and IL-6-mediated inflammatory signaling in aggressive breast cancer phenotypes (Hailemichael et al., 2022).

Selectivity index analysis demonstrated that both venoms preferentially targeted cancer cells over normal WI-38 fibroblasts. Scorpion venom showed high selectivity toward breast and colorectal cancer cells, consistent with membrane-targeting and apoptosis-inducing mechanisms, whereas bee venom displayed moderate but consistent selectivity, particularly against A549 lung cancer cells, in line with the pro-apoptotic activity of bioactive compounds that preferentially disrupt cancer cell membranes and activate intrinsic apoptotic pathways, especially in tumors with altered membrane composition and ion channel expression (Holford et al., 2018; Arcangeli and Becchetti, 2017). The lower selectivity of scorpion venom in A549 cells highlights tumor-specific resistance and underscores the cell line–dependent nature of venom bioactivity. This aligns with previous studies showing that bee venom induces apoptosis in lung cancer cells via mitochondrial dysfunction, caspase activation, and inhibition of NF-κB signaling (Oršolić, 2012; Son et al., 2007).

Despite the higher cytotoxicity of scorpion venom against MDA-MB-231 cells, qRT-PCR analysis was deliberately conducted on A549 lung adenocarcinoma cells. This choice was guided by the parallel use of WI-38 normal lung fibroblasts as the corresponding non-tumor control, enabling a tissue-matched evaluation of gene expression changes and selectivity within the same biological context. Such alignment between normal and cancer cell models allowed more accurate interpretation of venom-induced transcriptional modulation related to safety and therapeutic relevance. While extending qRT-PCR analysis to MDA-MB-231 cells would provide additional mechanistic insights, this was beyond the scope of the present study. Importantly, these experiments have already been conducted as part of an ongoing follow-up investigation and will be reported separately. Quantitative PCR analysis revealed venom-specific modulation of cancer-related pathways in A549 lung adenocarcinoma cells, with honeybee and scorpion venoms inducing distinct gene expression profiles. Immune-related genes showed differential regulation, as honeybee venom markedly upregulated IL-6 expression (14.8-fold) compared with scorpion venom (7.2-fold), suggesting a stronger activation of pro-inflammatory signaling that may contribute to its cytotoxic effects (Elfiky et al., 2019). In contrast, both venoms significantly downregulated IL-2 and IL-12 (p < 0.05), indicating suppression of selected immune regulatory pathways and potential modulation of tumor-associated immune responses (Ibrahim et al., 2020; Sriwidodo et al., 2025).

Apoptosis-related genes also displayed venom-specific regulation. Bax expression was reduced following both treatments, whereas Bcl-2 was upregulated (2.3-fold) by honeybee venom but downregulated by scorpion venom. These contrasting patterns suggest that honeybee venom may induce cancer cell death through alternative mechanisms, such as membrane disruption or caspase-independent pathways, while scorpion venom more effectively promotes apoptosis by inhibiting anti-apoptotic signaling (Moon et al., 2006; Heidari Esfahani and Doosti, 2021).

Both venoms significantly downregulated the angiogenic factor VEGF and the tumor suppressor P53 (p < 0.01), with honeybee venom exerting a stronger inhibitory effect on VEGF expression. This coordinated suppression may limit tumor growth and metastatic potential through combined anti-angiogenic and anti-proliferative actions (Lu et al., 2025). The observed cytotoxicity patterns align with venom-specific transcriptional responses, whereby honeybee venom predominantly influences immune and angiogenic pathways, while scorpion venom shows greater selectivity toward apoptotic regulation. Integrating these molecular insights with cytotoxicity data, molecular docking, and ADME predictions provides a robust framework for identifying venom-derived lead compounds with optimized anticancer efficacy and safety profiles (Andreu et al., 2022).

GC–MS analysis revealed that honeybee and scorpion venoms possess highly complex yet distinct chemical profiles that likely underlie their differential biological activities. Honeybee venom was dominated by a limited number of highly abundant compounds, particularly ethane, 1-chloro-1-fluoro (42.79%) and acetonitrile (24.97%), with lower levels of furazan, caprylic anhydride, and 1,3,5-triazine derivatives. The predominance of heterocyclic and halogenated constituents suggests enhanced solubility and cellular permeability, which may explain the broader modulation of angiogenic (VEGF) and immune-related (IL-6) pathways observed in gene expression analyses (Mabunda et al., 2025; Delgado-Prudencio et al., 2022).

In contrast, scorpion venom exhibited a more evenly distributed composition enriched in reactive low-molecular-weight compounds, including methyl isocyanide (28.93%), 3-butyn-1-ol (20.74%), and cumulatively abundant allene derivatives (∼25%), alongside minor components such as cis-aconitic anhydride and N-ethyl-N′-nitroguanidine. This chemically diverse mixture of isocyanides, alkynols, and nitroguanidines is consistent with the higher selective cytotoxicity and pronounced apoptotic induction observed in A549 and MDA-MB-231 cells (Zarei and Kazemi Noureini, 2017; Jung et al., 2014; Nosouhian et al., 2024).

Most identified compounds from both venoms exhibited low molecular weights, particularly below 150 g/mol, providing favorable scaffolds for lead optimization. Such structural simplicity supports chemical modification while maintaining acceptable pharmacokinetic properties, in line with fragment-based drug discovery strategies aimed at improving selectivity and efficacy (Utkin, 2021). Additionally, the consistent and favorable hydrogen bond donor/acceptor profiles across compounds suggest strong and stable interactions within protein active sites, which correlates with enhanced docking performance and higher hit discovery rates *in silico* (Tahiroğlu et al., 2025; Lafnoune et al., 2022). The purpose of the GC-MS analysis was to identify volatile and semi-volatile organic compounds that are rarely investigated in venom studies but may contribute to overall bioactivity. These small molecules may act as cytotoxic agents, membrane-disrupting compounds, or synergistic enhancers of peptide toxins. The compounds detected by GC-MS (e.g., fatty acids, esters, and alkanes) are unlikely to be the sole drivers of the observed anticancer effects. Instead, a synergistic model in which these small molecules may modulate tumor cell membrane properties or the local microenvironment, thereby facilitating the activity of venom peptides.

Recent computational studies further support the therapeutic relevance of venom-derived small molecules, demonstrating strong binding affinities toward leukemia-associated targets, including tyrosine kinases, topoisomerases, and apoptosis-regulating enzymes (Saragatsis et al., 2025). Here, the approach of molecular docking simulation of honeybee- and scorpion-venom–derived compounds against key cancer-related proteins revealed several ligands with strong binding affinities, notably palmitin, 2-mono-, caprylic anhydride, and 1,3,5-triazine, 2,4,6-tris(cyanomethoxy). These compounds showed stable interactions with cytokines (IL-6), angiogenic regulators (VEGFR), and apoptosis-associated proteins (Tp53, Bax, and BCL-2), indicating their capacity to simultaneously modulate multiple oncogenic pathways (Erdoğan et al., 2025; Saravanan et al., 2023; Mansour et al., 2021). The higher binding affinity between these ligands and receptors energetically favorable interactions suggest a high degree of complex stability and a potential role in promoting apoptotic signaling while attenuating pro-inflammatory pathways, consistent with previous reports on the multitarget activity of small venom-derived molecules (Nurhayati et al., 2022; Ochoa-Mosquera et al., 2024).

Compounds with intermediate activity, such as cis-aconitic anhydride and N-ethyl-N′-nitroguanidine exhibited docking scores between −4.3 and −5.6 kcal/mol, suggesting potential synergistic roles in multi-ligand therapeutic strategies (Seliem, 2025). Across all ligands, complex stabilization was primarily mediated by a combination of hydrogen bonding and hydrophobic interactions, reinforcing the importance of balanced polar and non-polar contacts in achieving selective and stable protein binding (Devi and Jayaraman, 2025; Truong et al., 2023).

Consistent with our molecular docking simulations aimed at predicting the lead potential of venom-derived bioactive compounds, several studies have highlighted the therapeutic relevance of natural molecules targeting apoptotic and immune-related proteins. Rath et al. (2021) demonstrated that compounds from *Moringa oleifera*, particularly quercetin, bind strongly to p53 (−6.72 kcal/mol), while Suwitono and Sulastri (2022) reported that vitamins such as folic acid (B9) and ergocalciferol (D2) exhibit high affinities toward multiple interleukins (−7.43 to −6.72 kcal/mol). These findings support the validity of our *in silico* approach and underscore the relevance of targeting immune and apoptotic pathways in anticancer drug discovery.

To our knowledge, this is the first study to systematically demonstrate the anticancer potential of bioactive compounds derived from honeybee and scorpion venoms using integrated cytotoxicity, selectivity, gene expression, and molecular docking analyses. Notably, although the most active compounds were present at relatively low abundances in the venoms, they exhibited stronger binding affinities to key protein targets than more abundant constituents, highlighting their potential as lead molecules. This observation emphasizes the importance of activity-based prioritization over compositional dominance in venom-based drug discovery.

Overall, the convergence of cytotoxicity, selectivity indices, transcriptional modulation, and docking data supports the role of venom-derived compounds as multitarget anticancer agents capable of modulating apoptosis, inflammation, and angiogenesis. The higher potency of scorpion venom, together with the more favorable safety profile of honeybee venom, reveals distinct yet complementary therapeutic advantages. Integrating these biological outcomes with physicochemical characterization, Lipinski filtering, and molecular docking provides a robust framework for prioritizing venom-derived lead compounds for further preclinical optimization and experimental validation (Aziz et al., 2022; Ramesh et al., 2025). The moderate to high binding affinities observed for specific honeybee and scorpion venom molecules suggest plausible interactions with the target receptors. However, docking results do not predict whether these interactions lead to gene upregulation or downregulation; they only indicate binding potential. Therefore, further experimental studies are required to elucidate the underlying molecular mechanisms.

## 5 Conclusion

In conclusion, this study demonstrates that honeybee and scorpion venoms possess distinct, yet complementary anticancer properties mediated through multi-target mechanisms. Scorpion venom exhibited stronger cytotoxic and pro-apoptotic effects, consistent with its higher affinity toward apoptotic regulators such as BCL-2 and Bax, but showed increased toxicity toward normal WI-38 fibroblasts. In contrast, honeybee venom displayed moderate cytotoxicity with a more favorable safety profile and pronounced immunomodulatory and anti-angiogenic effects, supported by its higher affinity for IL-6 and VEGFR-2 and confirmed by qRT-PCR analysis. The integration of GC–MS profiling, molecular docking, cytotoxicity assays, and gene expression analysis provides a comprehensive framework linking venom chemical composition to functional biological outcomes. By directly comparing both venoms across multiple cancer and normal cell lines, this work offers new insight into venom-specific selectivity patterns and highlights venom-derived bioactive compounds as promising multi-target anticancer candidates especially toward A549 cells. These findings lay a strong foundation for future *in vivo* validation and for the isolation and optimization of lead compounds with improved efficacy and selectivity.

## Data Availability

The datasets presented in this study can be found in online repositories. The names of the repository/repositories and accession number(s) can be found in the article/Supplementary Material.
