# Supplementary material for: A comparison of honeybee and scorpion venoms as anticancer agents against three different cancer cell lines: lung, colon, and breast cancer
Source: Front Toxicol. 2026 Feb 25;8:1756933. doi: 10.3389/ftox.2026.1756933 (PMC12975568; doi:10.3389/ftox.2026.1756933)
Supplement: Supplementary file 1 [file Table1.docx]

Supplementary Material

**Supplementary Table 1**. Evaluation of honeybee and scorpion venom–derived bioactive compounds according to Lipinski’s Rule of Five using the SwissADME server

| **Ligand** | **Lipinski’s Rule of Five** | | | | | |
| --- | --- | --- | --- | --- | --- | --- |
|  | **iLogP <5** | **Molecular wight (g/mol) <500** | **Hydrogen acceptor <10** | **Hydrogen donor <5** | **Drug-likeness Lipinski’s rule follows** | **Violation** |
|  | **Compounds identified in Honeybee Venom** | | | | | |
| Propadiene | 1.45 | 40.06 | 0 | 0 | yes | 0 |
| 2-Hexynoic acid | 1.44 | 112.13 | 2 | 1 | yes | 0 |
| Furazan | 1.06 | 70.05 | 3 | 0 | yes | 0 |
| -Methyl-1,5-(4H)-dihydropyrido-(2,3-b)1,4-diazepine-4-one | 1.43 | 175.19 | 2 | 2 | yes | 0 |
| Ethane, 1-chloro-1-fluoro | 1.40 | 82.50 | 1 | 0 | yes | 0 |
| Acetonitrile | 0.84 | 41.05 | 1 | 0 | yes | 0 |
| 2-Butanone | 1.42 | 72.11 | 1 | 1 | yes | 0 |
| Butanedioic acid | 0.32 | 118.09 | 4 | 2 | yes | 0 |
| 4-Spirohexanone, 5,5-dichloro- $$ 5,5-Dichlorospiro[2.3]hexan-4-one | 1.60 | 165.02 | 1 | 0 | yes | 0 |
| Isobutyric acid, allyl ester | 2.30 | 128.17 | 2 | 0 | yes | 0 |
| 2-Pentanone | 1.61 | 86.13 | 1 | 0 | yes | 0 |
| cis-Aconitic anhydride | 0.34 | 156.09 | 5 | 1 | yes | 0 |
| l-Felinine | 1.24 | 207.29 | 4 | 3 | yes | 0 |
| Palmitin, 2-mono | 4.50 | 330.50 | 4 | 2 | yes | 0 |
| 3-Ethyl-3-heptanol | 2.71 | 144.25 | 1 | 1 | yes | 0 |
| 2,5-Furandione, dihydro-3-methylene- | 0.86 | 112.08 | 3 | 0 | yes | 0 |
| cis-Aconitic anhydride | 0.34 | 156.09 | 5 | 1 | yes | 0 |
| 1,3,5-Triazine, 2,4,6-tris(cyanomethoxy | 1.26 | 246.18 | 9 | 0 | yes | 0 |
| 4-Spirohexanone, 5,5-dichloro- | 1.60 | 165.02 | 1 | 0 | yes | 0 |
| Methanethioamide, N,N-dimethyl- | 1.59 | 89.16 | 0 | 0 | yes | 0 |
| n-Dodecyl methacrylate | 4.09 | 254.41 | 2 | 0 | yes | 0 |
| Caprylic anhydride | 3.91 | 270.41 | 2 | 0 | yes | 0 |
| **Compounds identified in Scorpion Venom** | | | | | | |
| Allene | 1.45 | 40.06 | 0 | 0 | yes | 0 |
| Methyl isocyanide | 0.00 | 41.05 | 1 | 0 | yes | 0 |
| 3-Butyn-1-ol | 1.45 | 70.09 | 1 | 1 | yes | 0 |
| N-Ethyl-N'-nitroguanidine | 0.47 | 132.12 | 3 | 2 | yes | 0 |
| Sulphonyl diacetonitrile | 0.16 | 144.15 | 4 | 0 | yes | 0 |
| 1,2-Propadiene-1,3-dione | 0.32 | 68.03 | 2 | 0 | yes | 0 |
| 2-Methylaminomethyl-1,3-dioxolane | 1.86 | 117.15 | 3 | 1 | yes | 0 |
| cis-Aconitic anhydride | 0.34 | 156.09 | 5 | 1 | yes | 0 |
| N-Methoxymethyl-N-methylformamide | 1.50 | 103.12 | 2 | 0 | yes | 0 |
| Ethanamine, 2-(2,6-dimethylphenoxy)-N-methyl- | 2.68 | 179.26 | 2 | 0 | yes | 0 |
| 1,4,7,10-Tetraoxacyclododecane | 2.14 | 176.21 | 4 | 0 | yes | 0 |
| p-Dioxane, methylene- | 1.73 | 100.12 | 2 | 0 | yes | 0 |

**Supplementary Table 2.** Interaction affinity scores (kcal/mol), RMSD values (Å), and molecular interactions of bioactive molecules derived from bee and scorpion venoms with active-site residues of target protein receptors.

| **Ligand** | **interaction affinity score ( kcal/mol), RMSDValue (Å)** | | | | | | |
| --- | --- | --- | --- | --- | --- | --- | --- |
|  | **Tp53** | **Il2** | **Il12** | **Bax** | **BCL-2** | **IL6** | **VEGR** |
|  | **Compounds identified in Honeybee Venom** | | | | | |  |
| Propadiene | -3.2, 1.46 | -3.30, 0.6 | -3.55, 2.2 | -3.24, 1.1 | -3.41, 1.4 | -3.1, 1.5 | -3.3, 1.7 |
| 2-Hexynoic acid | -5.30, 1.48 | -4.45, 1.5 | -5.06, 0.6 | -4.83, 1.7 | -4.59, 1.5 | -4.9, 1.6 | -4.7, 1.2 |
| Furazan | -3.7, 1.64 | -2.91, 1.9 | -4.12, 1.0 | -3.74, 1.7 | -3.74, 0.7 | -3.6, 1.9 | -3.6, 1.9 |
| -Methyl-1,5-(4H)-dihydropyrido-(2,3-b)1,4-diazepine-4-one | -4.81, 0.71 | -4.71, 1.1 | -3.88, 1.1 | -5.15, 0.9 | -4.6, 1.6 | -5.2, 1.5 | -5.3, 1.3 |
| Ethane, 1-chloro-1-fluoro | -3.38, 1.7 | -3.34, 0.8 | -3.91, 1.2 | -3.49, 0.6 | -3.6, 1.2 | -3.5, 1.1 | -3.5, 0.73 |
| Acetonitrile | -3.13, 0.7 | -3.27, 1.1 | -3.67, 1.4 | -3.04, 1.2 | -3.2, 1.3 | -3.4, 0.71 | -3.3, 1.0 |
| 2-Butanone | -3.84, 0.5 | -4.00, 0.5 | -4.00,0.5 | -4.27, 0.5 | -3.9, 1.8 | -4.0, 0.49 | -4.13, 0.56 |
| Butanedioic acid | -5.24, 1.4 | -4.08, 2.0 | -5.08, 0.9 | -4.62, 0.9 | -4.0, 1.0 | -4.3, 1.3 | -4.19, 2.3 |
| 4-Spirohexanone, 5,5-dichloro- $$ 5,5-Dichlorospiro[2.3]hexan-4-one | -4.24, 1.7 | -4.18, 1.2 | -3.60, 0.9 | -4.38, 1.0 | -4.63, 0.8 | -4.3, 1.0 | -4.8, 1.60 |
| Isobutyric acid, allyl ester | -4.46, 1.3 | -4.60, 0.8 | -5.00, 0.5 | -4.82, 1.4 | -4.91, 0.8 | -4.7, 1.4 | -4.2, 1.3 |
| 2-Pentanone | -4.05, 1.2 | -4.24, 1.2 | -4.68, 0.6 | -4.28, 1.0 | -4.44, 0.9 | -4.3, 0.82 | -4.3, 0.8 |
| cis-Aconitic anhydride | -5.37, 1.5 | -4.34, 0.5 | -5.29, 0.5 | -5.28, 1.4 | -4.4, 0.7 | -4.8, 1.1 | -4.8, 1.2 |
| l-Felinine | -5.66, 1.3 | -4.62, 1.2 | -0.73, 1.0 | -5.16, 1.9 | -4.8, 1.2 | -5.7, 1.9 | -6.7 1.2 |
| Palmitin, 2-mono | -7.15,1.4 | -6.60, 1.1 | -+4.98, 1.9 | -6.48, 1.2 | -6.82, 1.2 | -6.9, 2.0 | -8.47 1.3 |
| 3-Ethyl-3-heptanol | -4.98, 1.6 | -4.73, 0.8 | -3.28, 1.3 | -4.59, 1.8 | -4.5 1.0 | -4.7, 0.88 | -5.6, 1.2 |
| 2,5-Furandione, dihydro-3-methylene- | -4.21, 1.6 | -4.21, 1.8 | -4.84, 1.3 | -4.57, 1.1 | -4.1 0.74 | -4.3, 1.8 | -4.2, 1.3 |
| 1,3,5-Triazine, 2,4,6-tris(cyanomethoxy | -6.45, 1.2 | -5.62, 1.3 | -0.89, 0.9 | -6.58, 1.7 | -5.65 1.2 | -5.9, 1.3 | -6.4, 1.3 |
| Methanethioamide, N,N-dimethyl- | -4.01, 1.1 | -3.55, 0.8 | -4.49, 1.5 | -4.12, 1.0 | -3.87 1.9 | -3.7, 1.7 | -3.8, 1.6 |
| n-Dodecyl methacrylate | -6.25, 1.4 | -5.8, 1.2 | +2.46, 1.4 | -6.58, 1.6 | -5.8 1.7 | -5.9, 1.8 | -6.9, 1.1 |
| Caprylic anhydride | -6.9, 1.7 | -6.1, 1.5 | -0.6, 2.2 | -6.7, 1.6 | -6.2 1.6 | -5.9, 1.8 | -7.9, 0.99 |
| **Compounds identified in Scorpion Venom** | | | | | | |  |
| Allene | -3.2 1.4 | -3.31, 0.6 | -3.5, 2.2 | -3.2, 0.27 | -3,6, 0.84 | -3.1, 0.6 | -3.3, 0.8 |
| Methyl isocyanide | -3.05 0.81 | -3.19, 0.5 | -3.5, 2.4 | -3.2, 1.5 | -3.1, 1.0 | -3.04, 2.2 | -2.9, 2.5 |
| 3-Butyn-1-ol | -4.1 2.1 | -4.08, 0.8 | -4.6, 0.8 | -4.08, 1.8 | -3.8, 1.2 | -4.04, 1.1 | -3.8, 1.7 |
| N-Ethyl-N'-nitroguanidine | -5.31 2.2 | -4.6, 1.6 | -5.3, 1.4 | -5.03, 1.6 | -4.5, 1.14 | -4.6, 1.3 | -4.7, 1.0 |
| Sulphonyl diacetonitrile | -4.7 2.1 | -4.1, 0.5 | -4.81, 1.07 | -4.11, 1.8 | -4.31, 1.09 | -4.7, 1.19 | -4.5, 0.5 |
| 1,2-Propadiene-1,3-dione | -3.8 2.1 | -3.6 2.0 | -4.15, 1.1 | -3.8, 0.59 | -3.6, 1.6 | -3.7, 1.5 | -3.7, 0.95 |
| 2-Methylaminomethyl-1,3-dioxolane | -4.05 1.4 | -4.17, 1.5 | -4.4, 0.86 | -4.4, 0.24 | -4.5, 1.1 | -4.6, 1.3 | -4.5, 1.4 |
| cis-Aconitic anhydride | -5.2 0.91 | -4.61, 1.1 | -5.3, 0.75 | -5.2, 1.3 | -4.34, 1.1 | -4.9, 0.53 | -4.8, 0.8 |
| N-Methoxymethyl-N-methylformamide | -4.5 1.6 | -4.01. 0.91 | -5.2, 1.1 | -4.3, 2.0 | -4.39, 0.74 | -4.3, 1.2 | -4.4, 0.6 |
| Ethanamine, 2-(2,6-dimethylphenoxy)-N-methyl- | -4.8 2.1 | -4.71, 0.8 | -1.1, 1.0 | -5.6, 0.80 | -4.8, 0.79 | -5.2, 1.1 | -6.3, 1.0 |
| 1,4,7,10-Tetraoxacyclododecane | -4.9 1.3 | -4.9, 0.9 | -3.07 1.3 | -5.03, 1.01 | -5.1, 1.2 | -5.1, 0.81 | -5.7, 1.2 |
| p-Dioxane, methylene- | -3.9 1.4 | -4.27, 1.7 | -4.7 0.5 | -4.18 0.89 | -4.08, 0.98 | -4.1, 1.0 | -4.3, 0.8 |

| **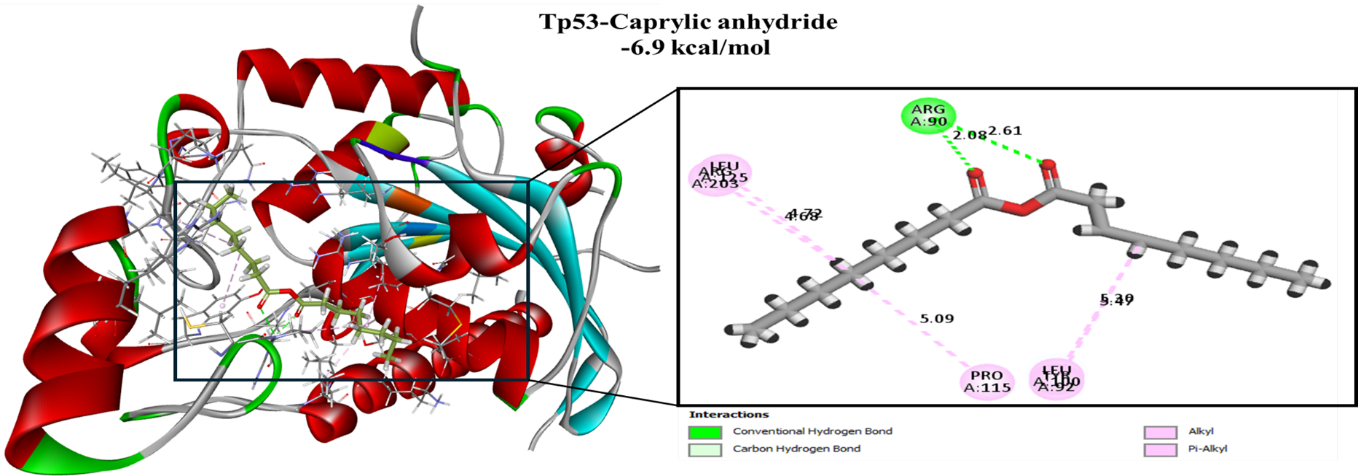** |
| --- |
| **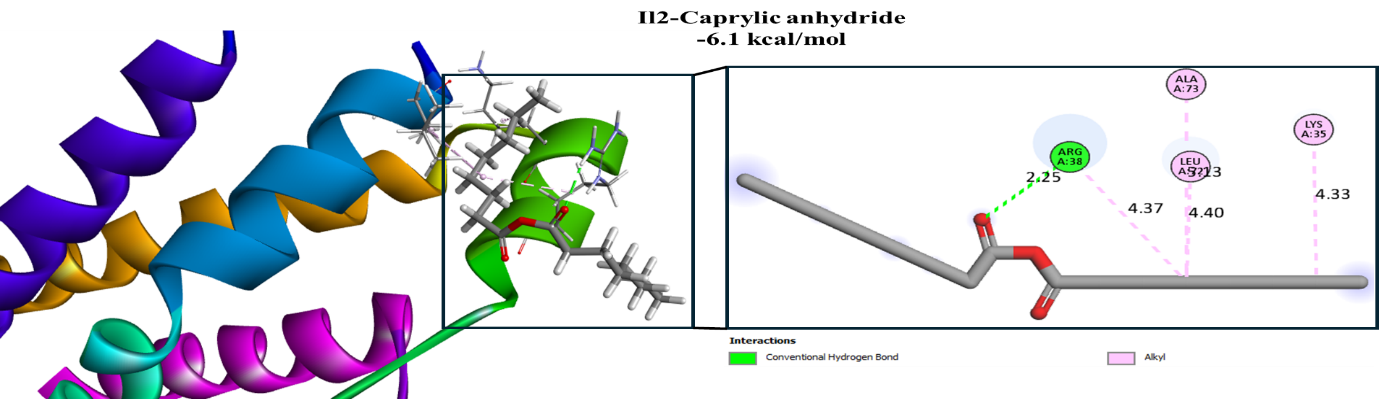** |
| **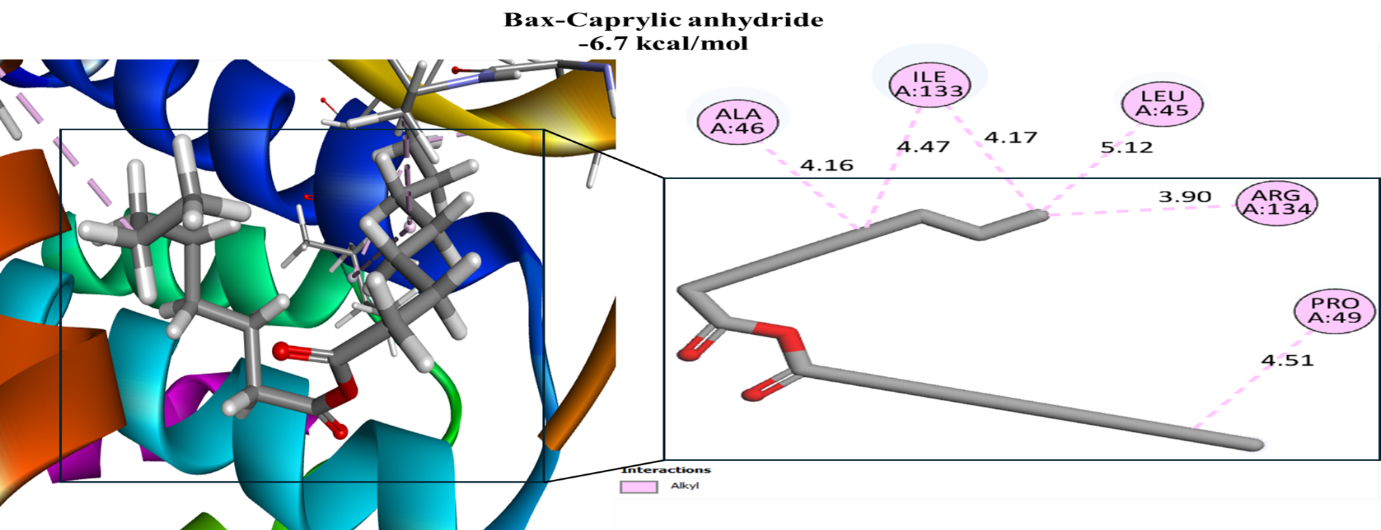** |
| **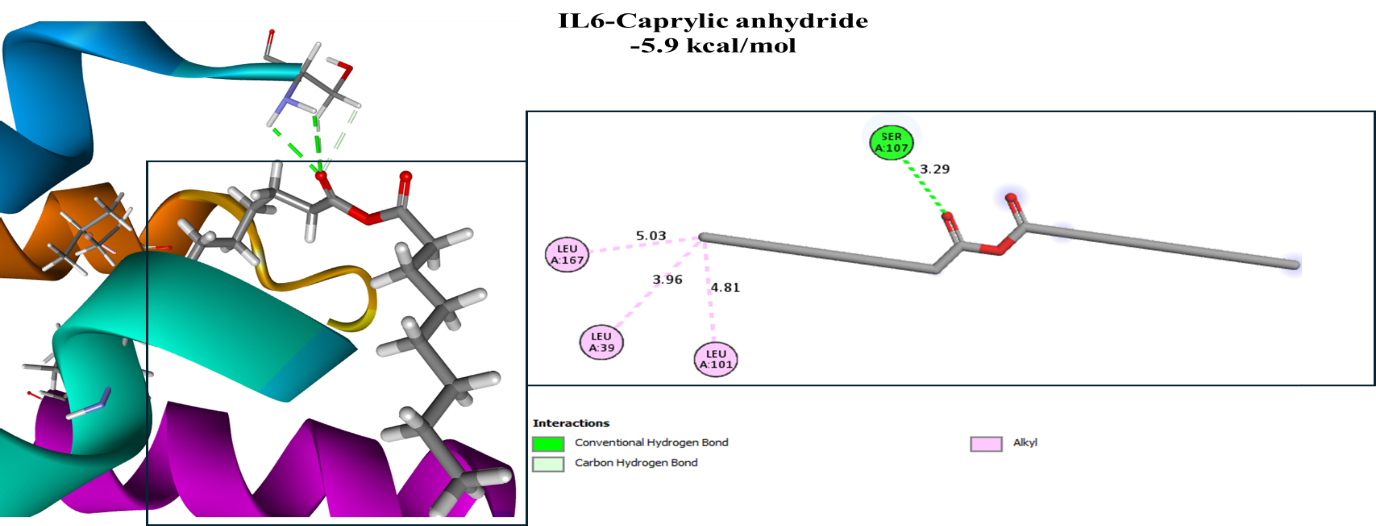** |
| **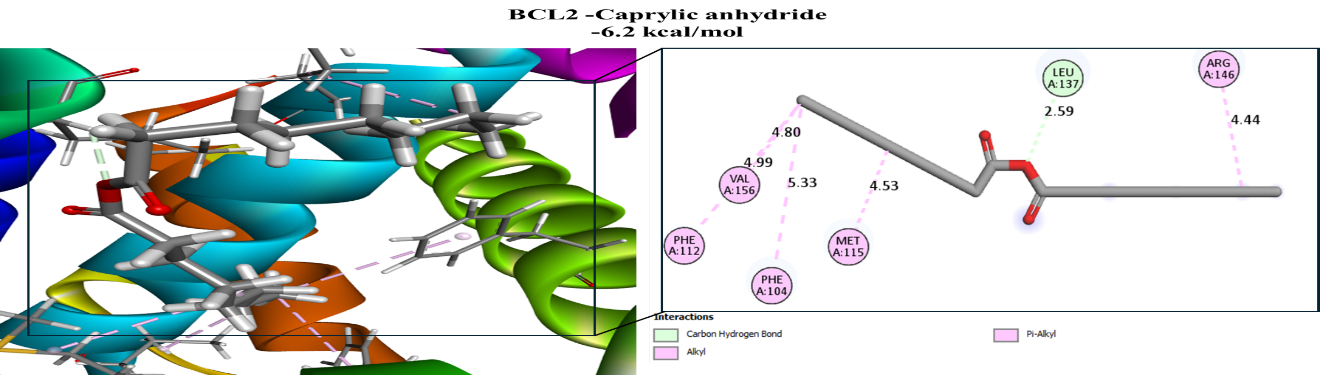** |
| **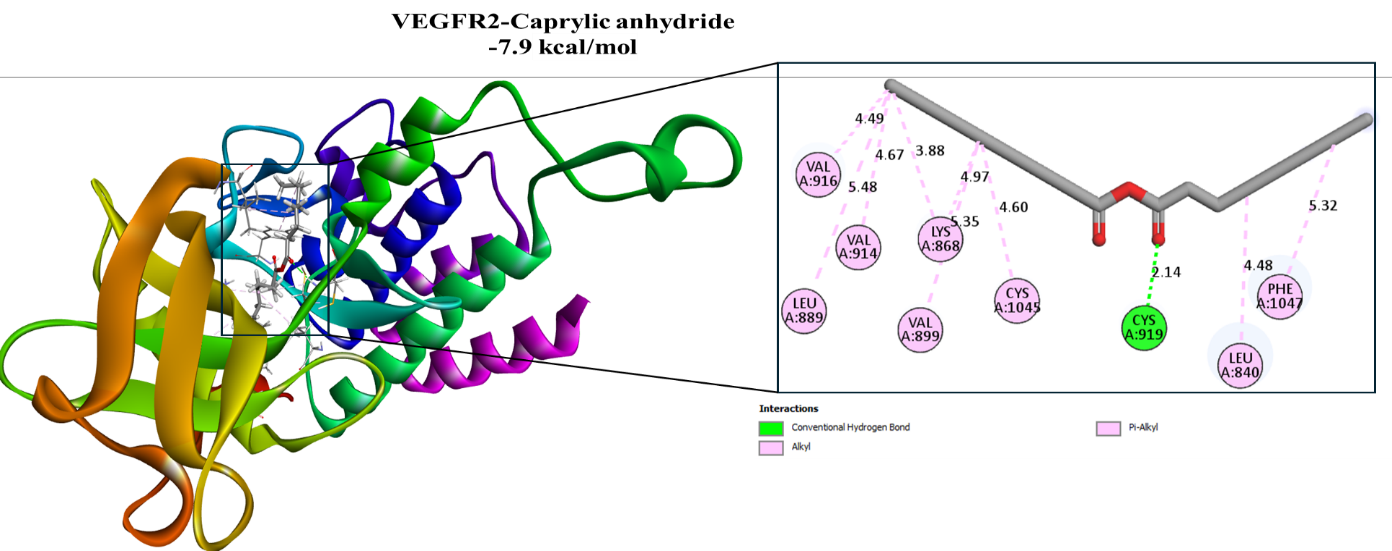** |
| **Supplementary Figure1:** Docking interactions of caprylic acid with active-site residues of target protein receptors. |

| 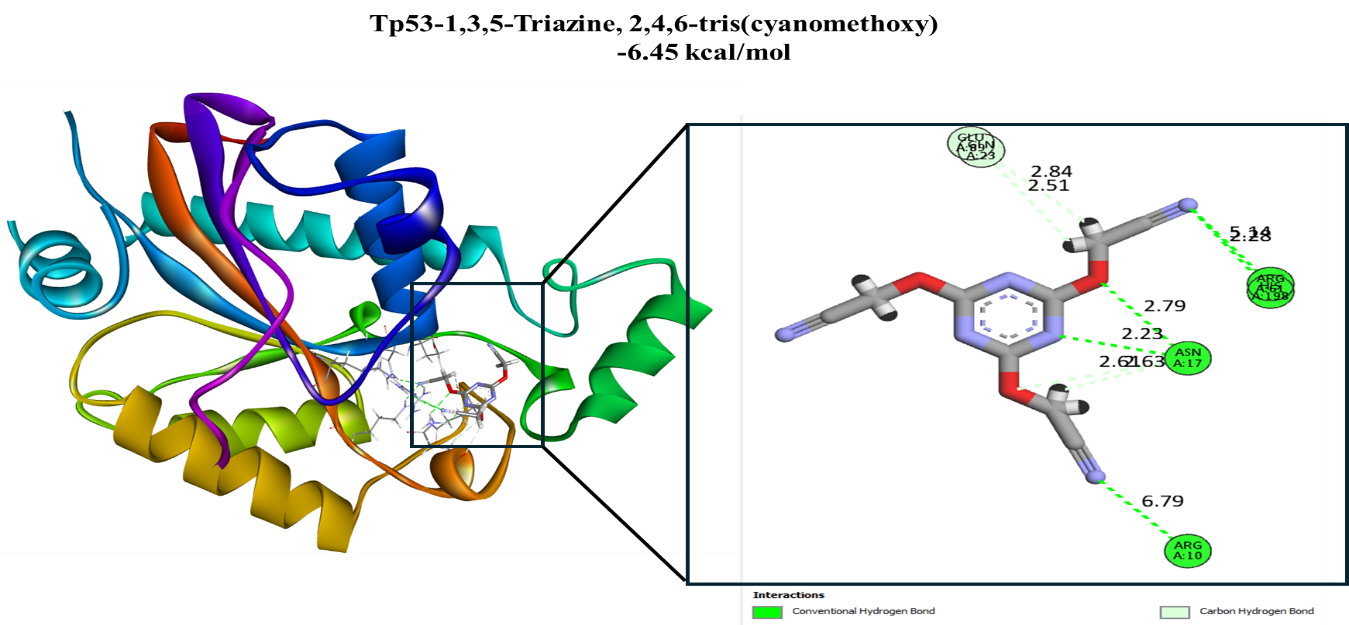 |
| --- |
| 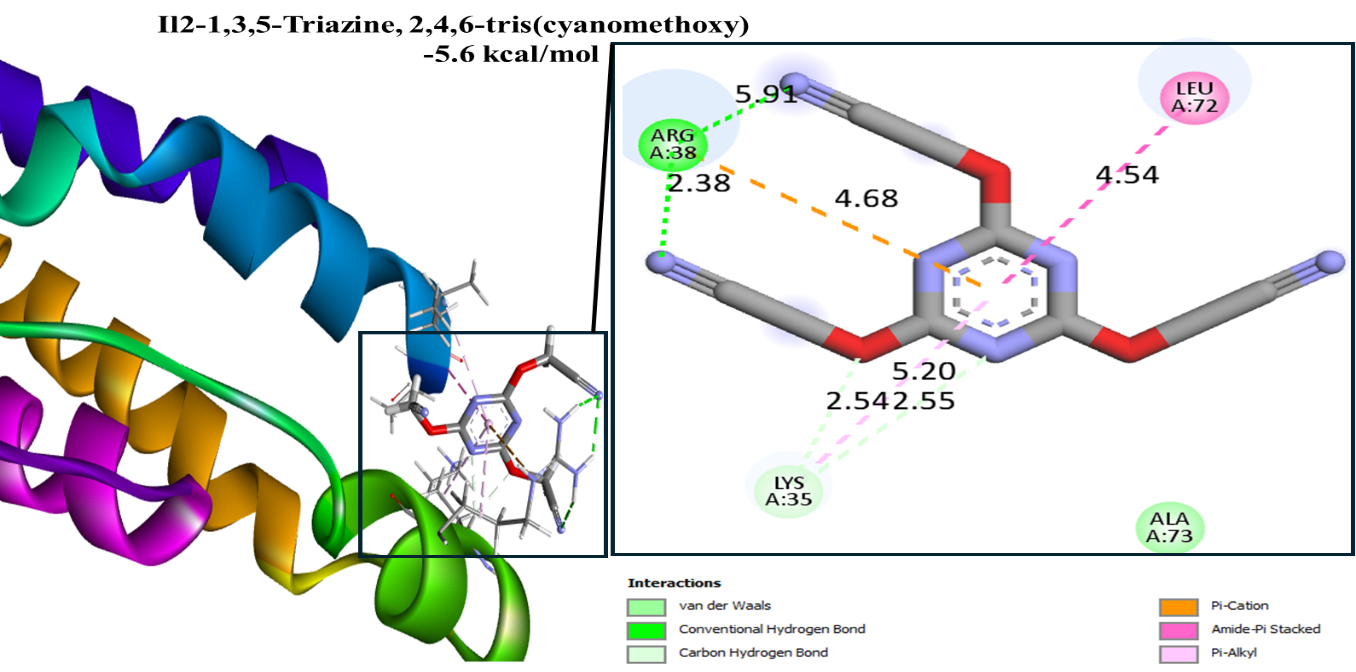 |
| 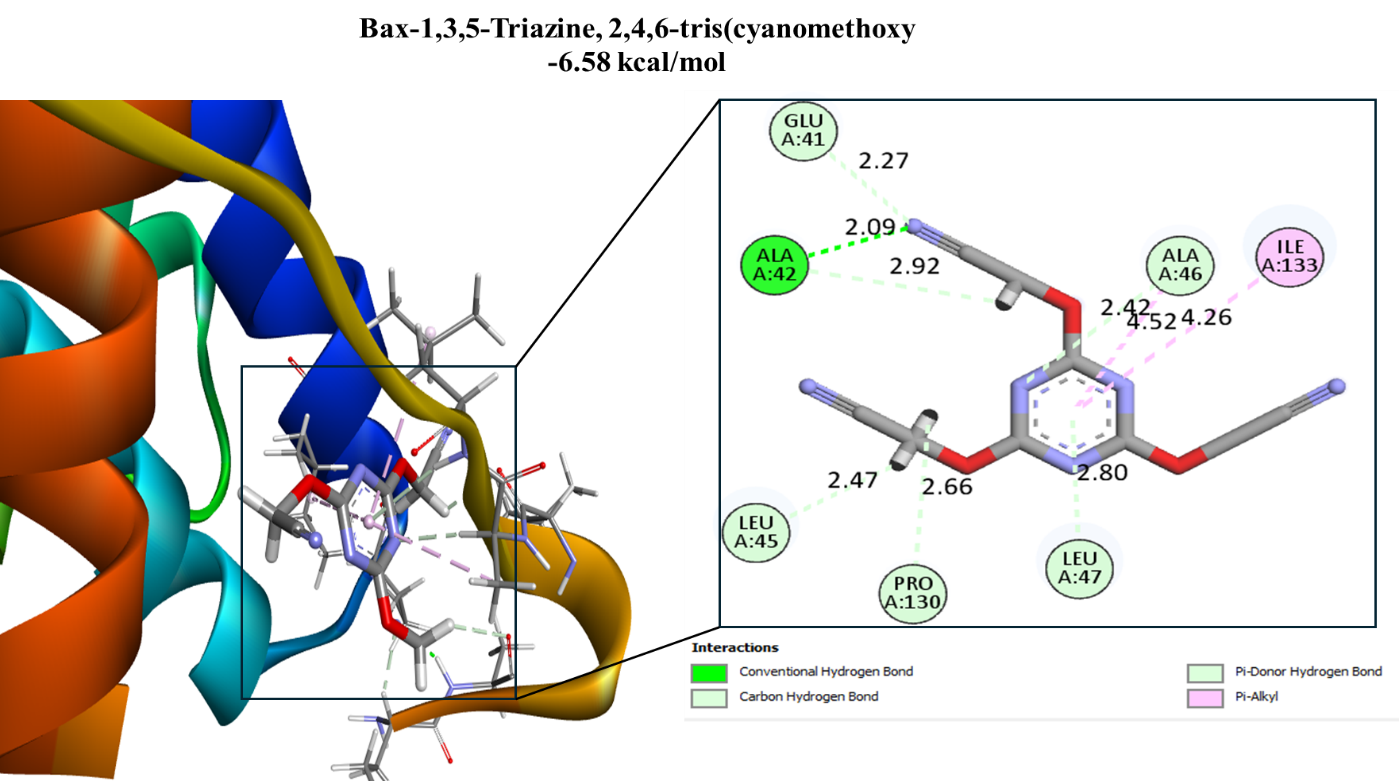 |
| 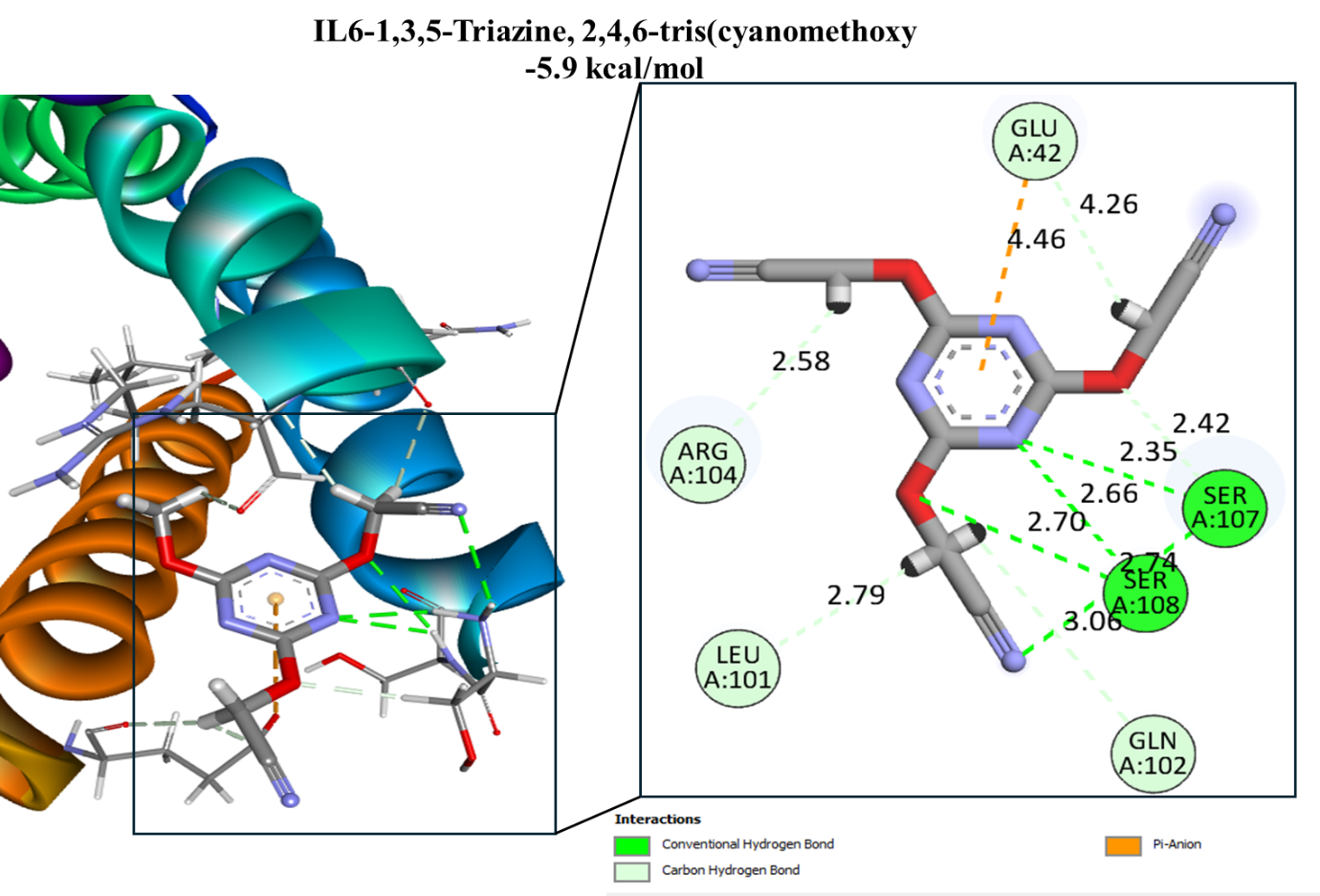 |
| 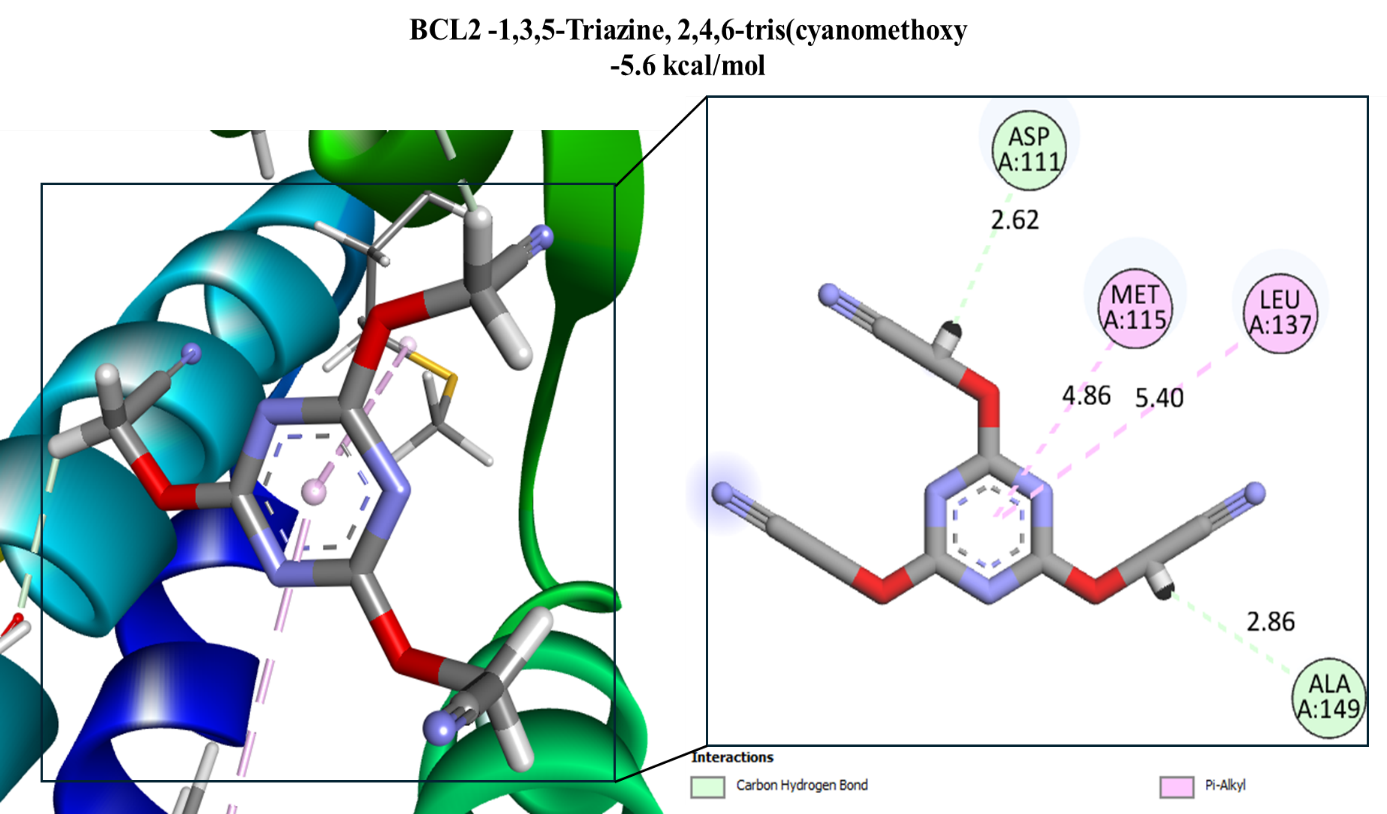 |
| 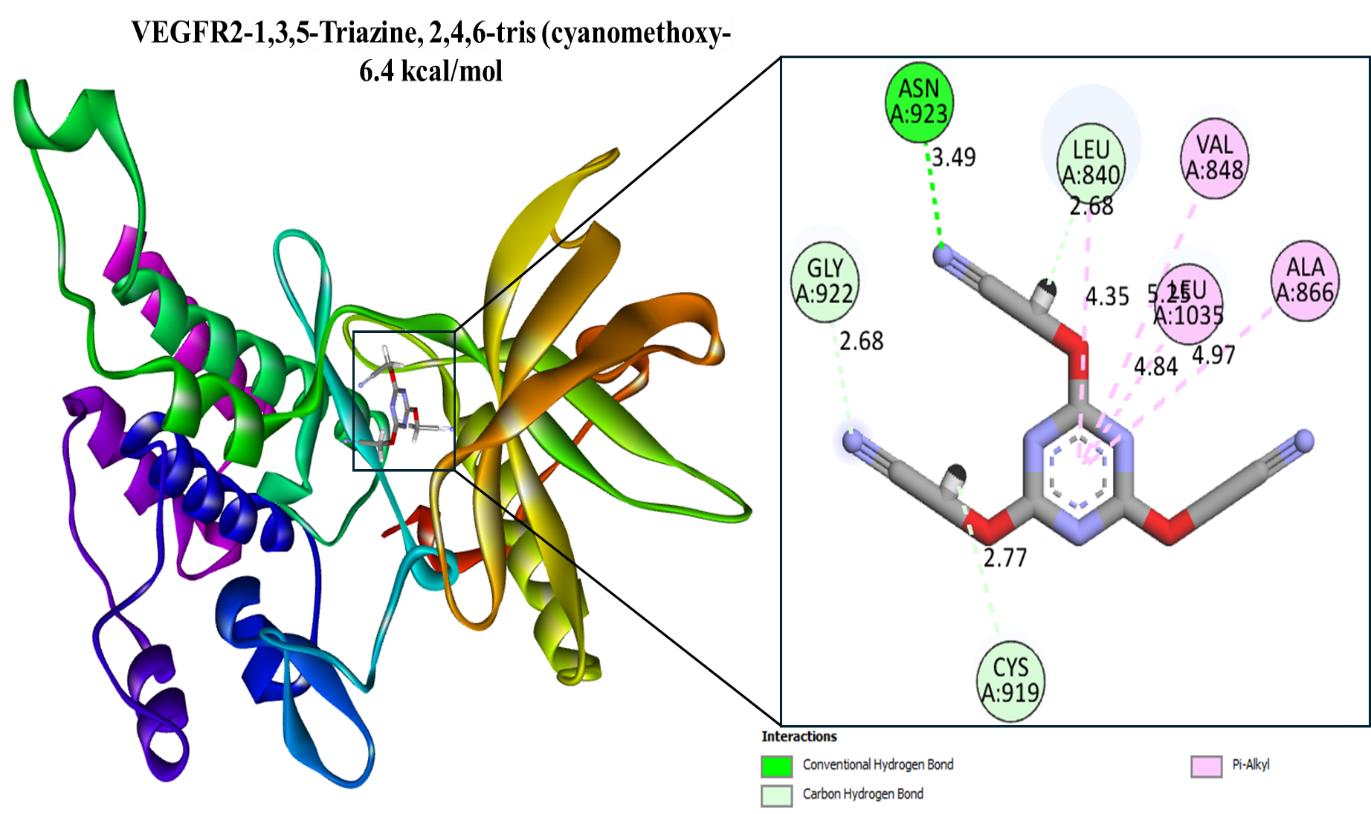 |
| **Supplementary Figure 2:** Docking interactions of 1,3,5-Triazine, 2,4,6-tris(cyanomethoxy with active-site residues of target protein receptors. |

| 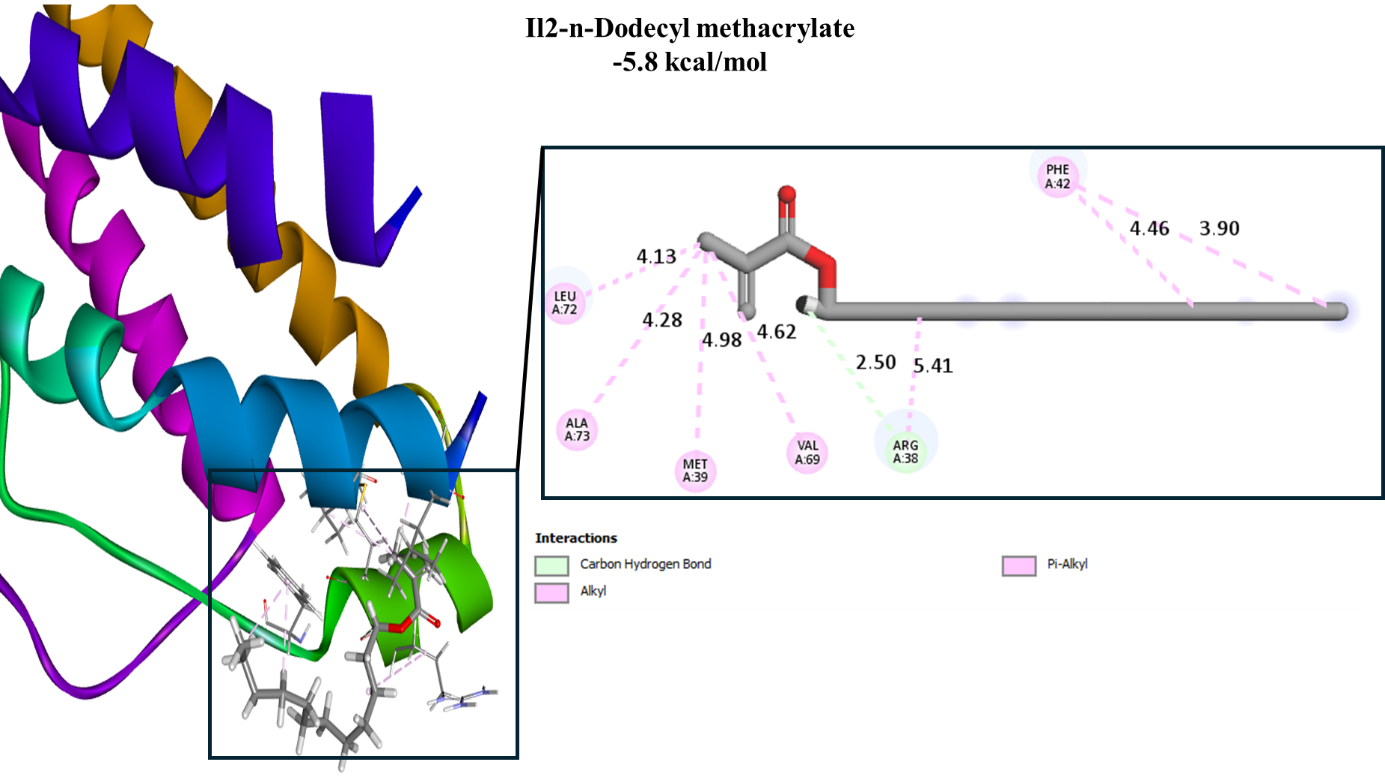 |
| --- |
| 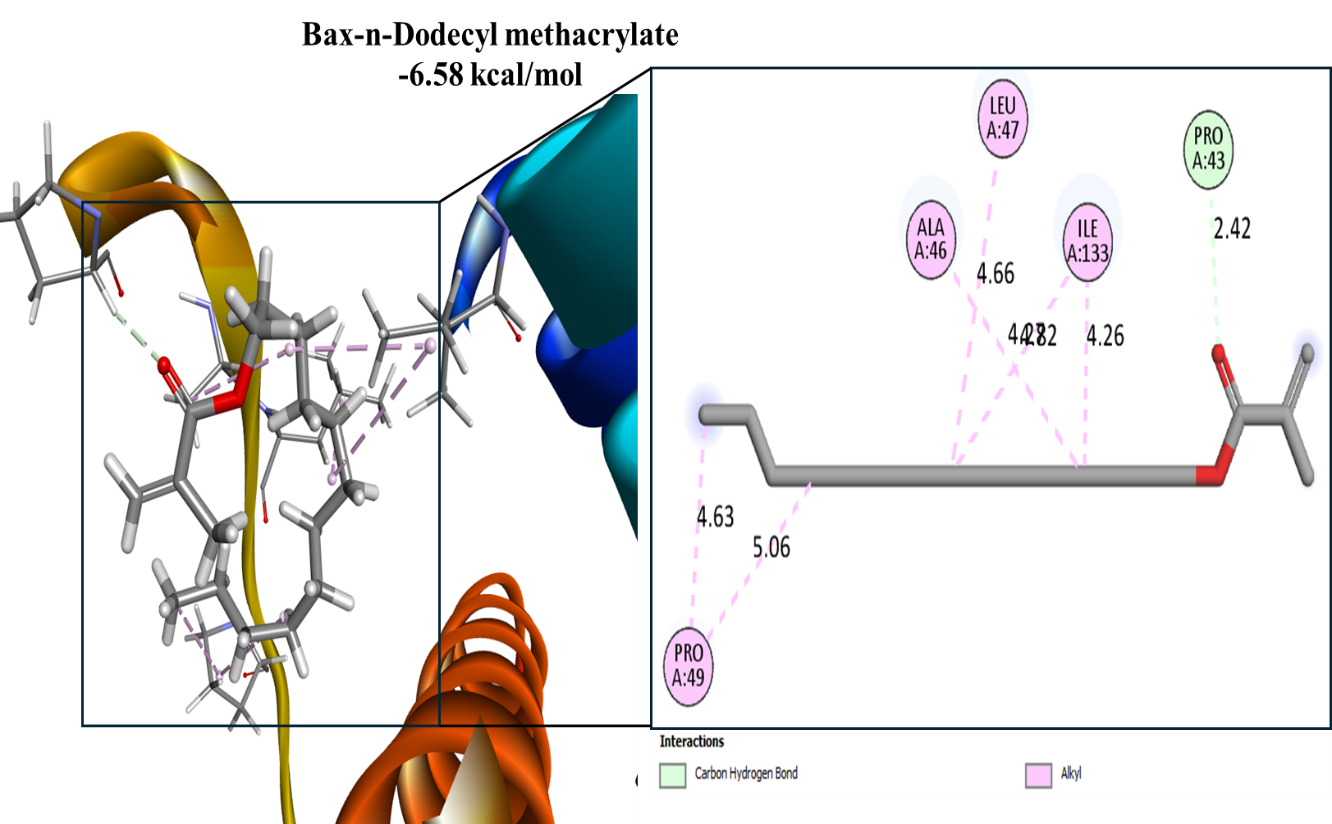 |
| 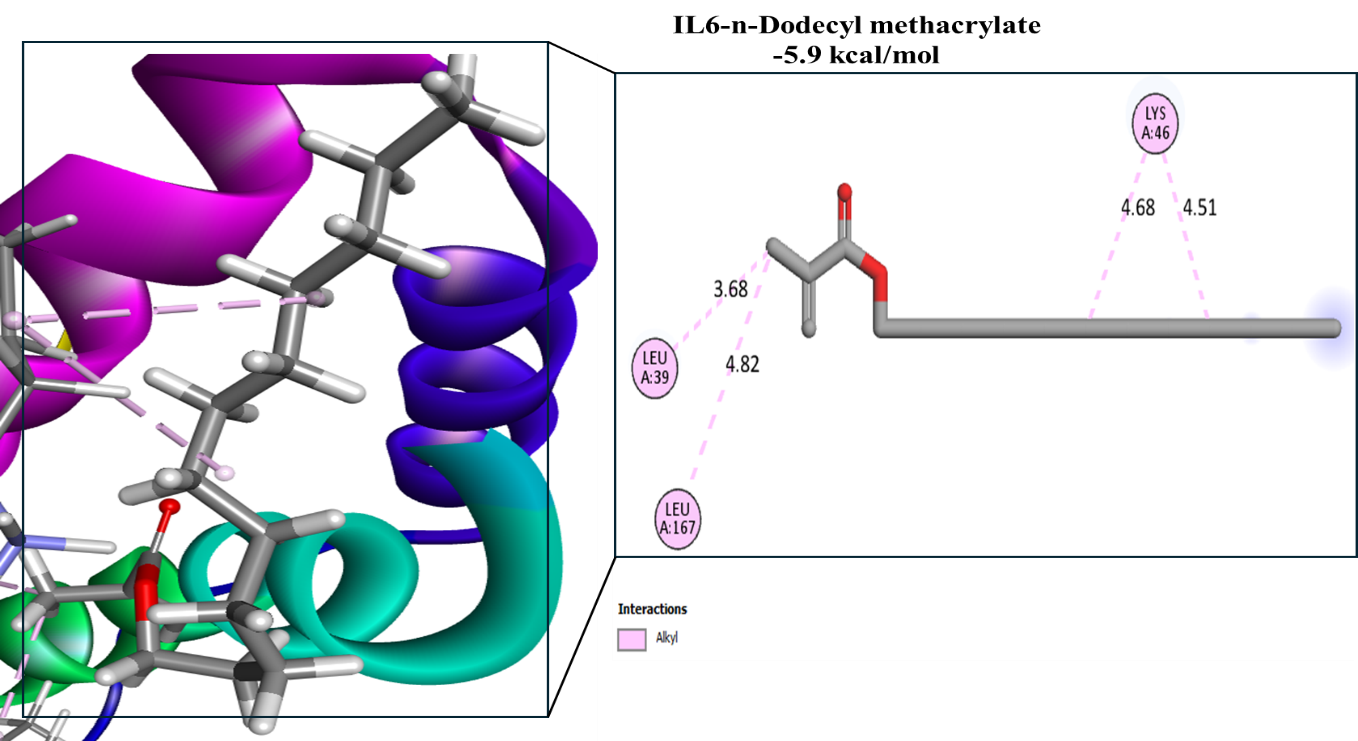 |
| 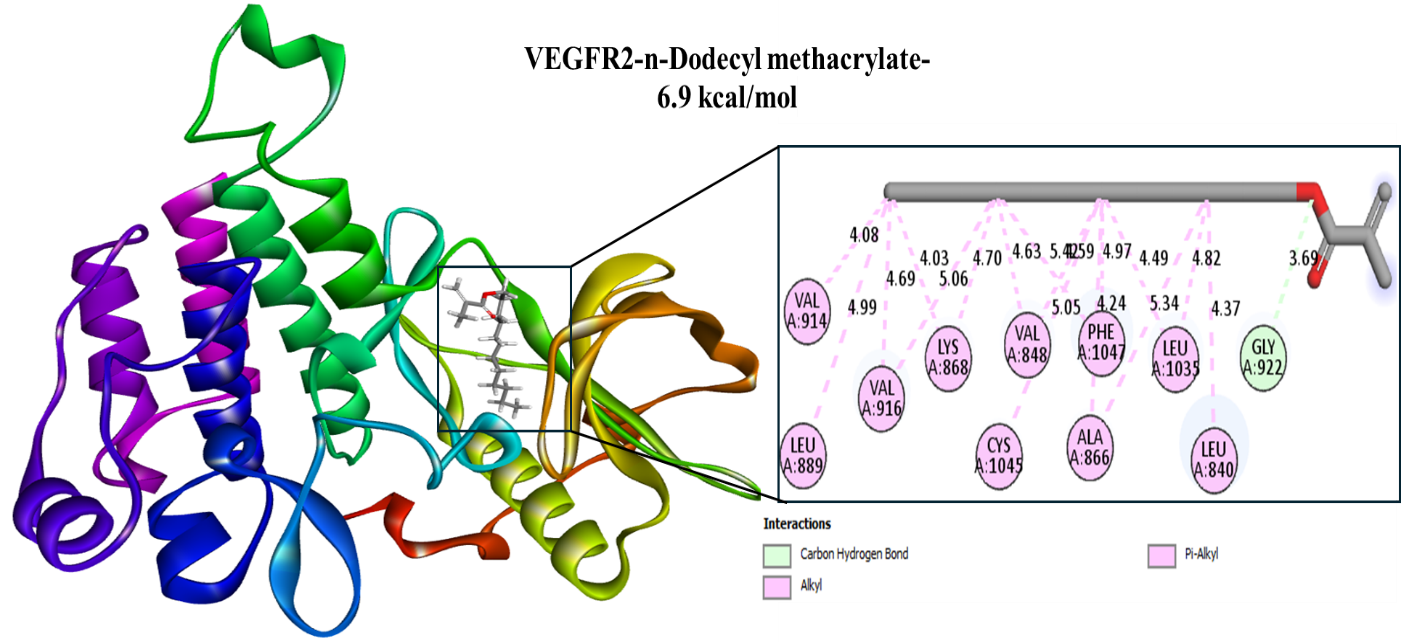 |
| **Supplementary Figure 3:** Docking interactions of n-Dodecyl methacrylate with active-site residues of target protein receptors |

| 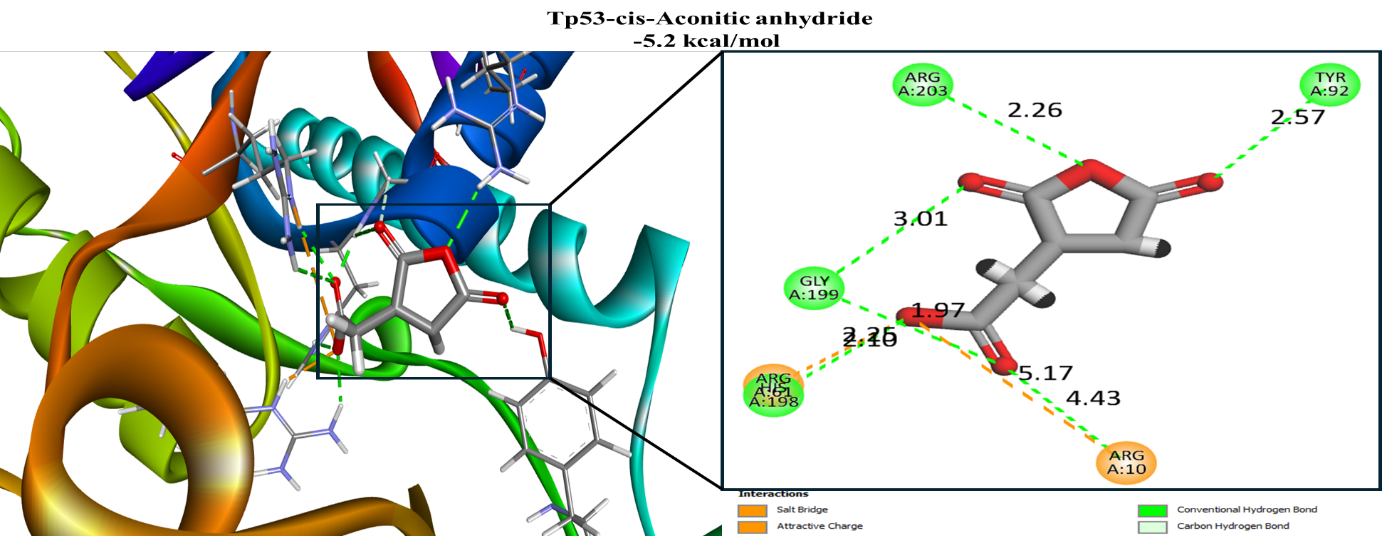 |
| --- |
| 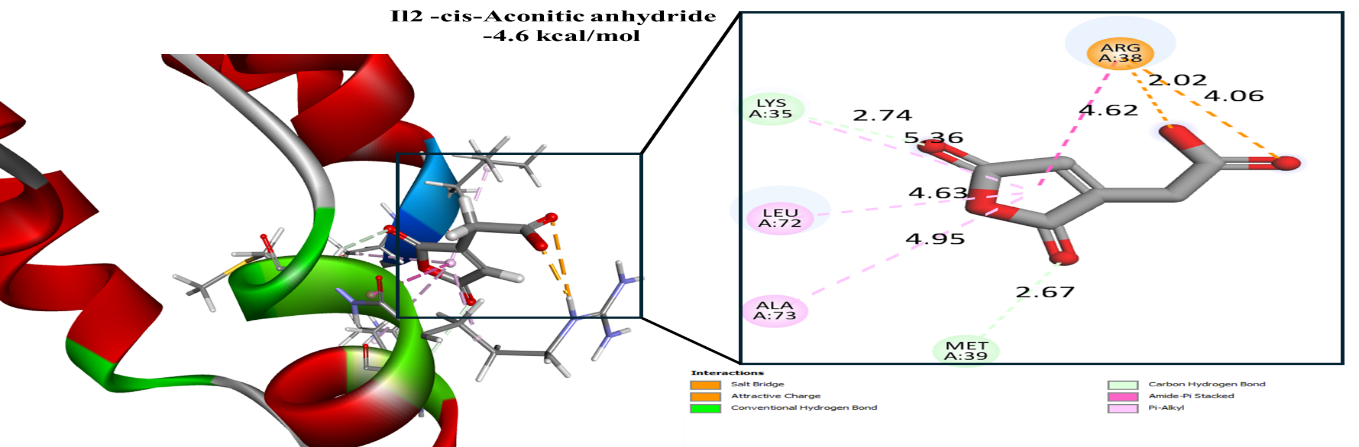 |
| 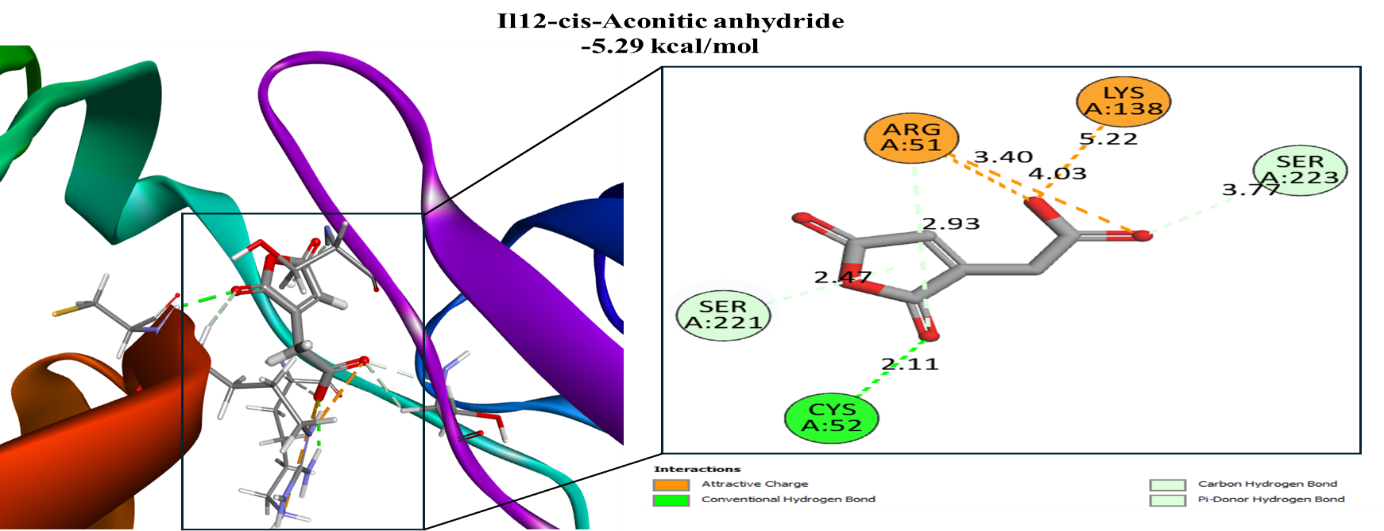 |
| 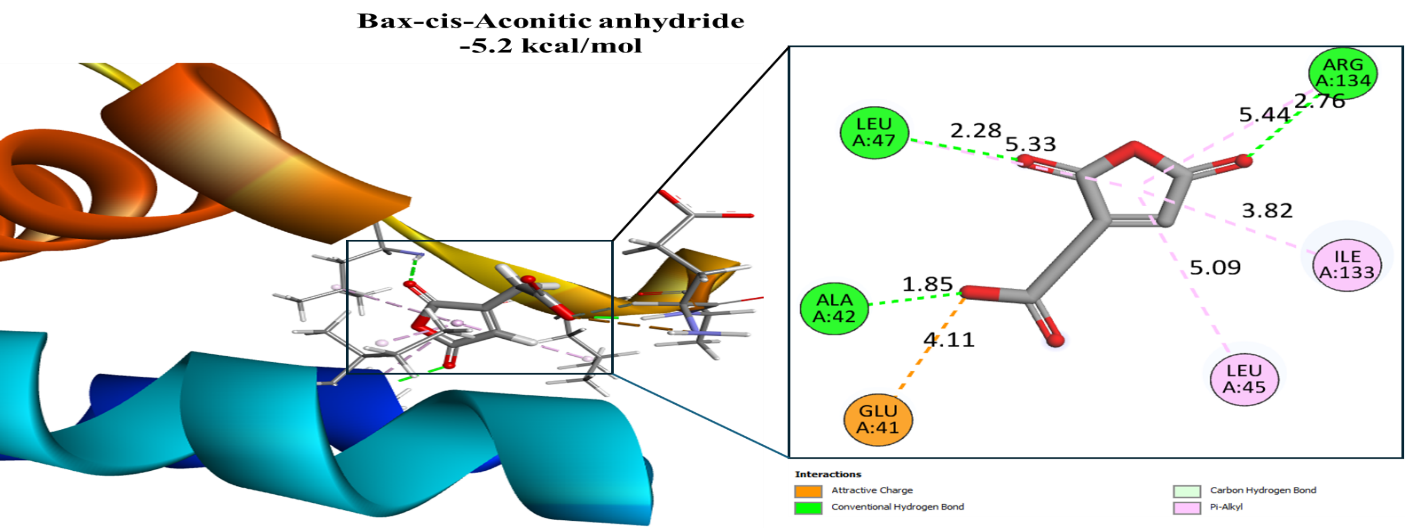 |
| 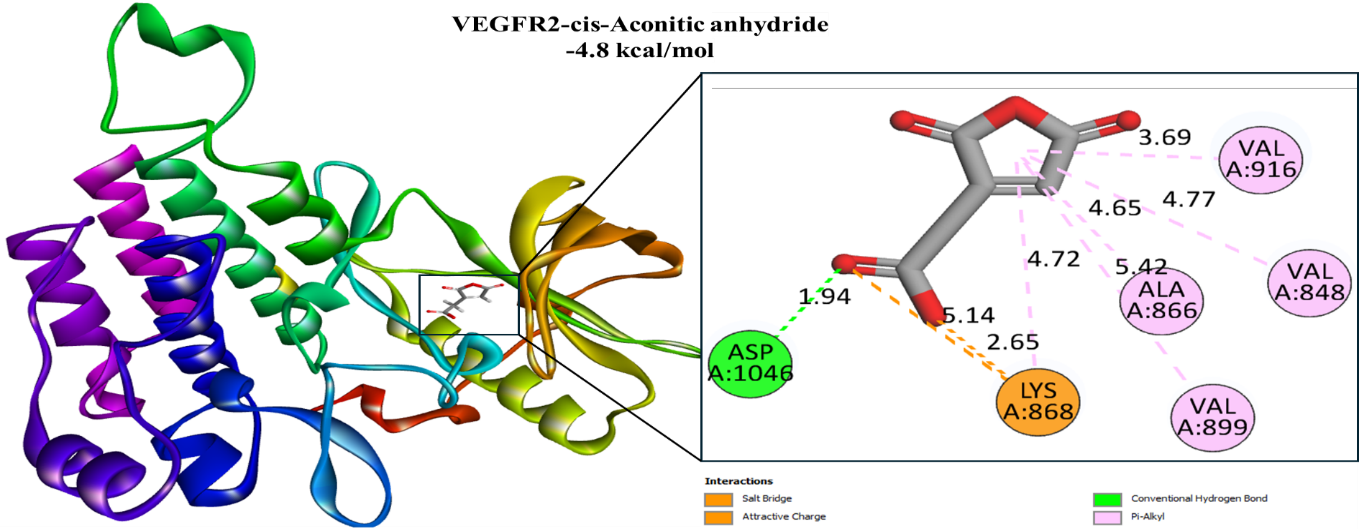 |
| **Supplementary Figure 4:** Docking interactions of cis-Aconitic anhydride with active-site residues of target protein receptors |

| 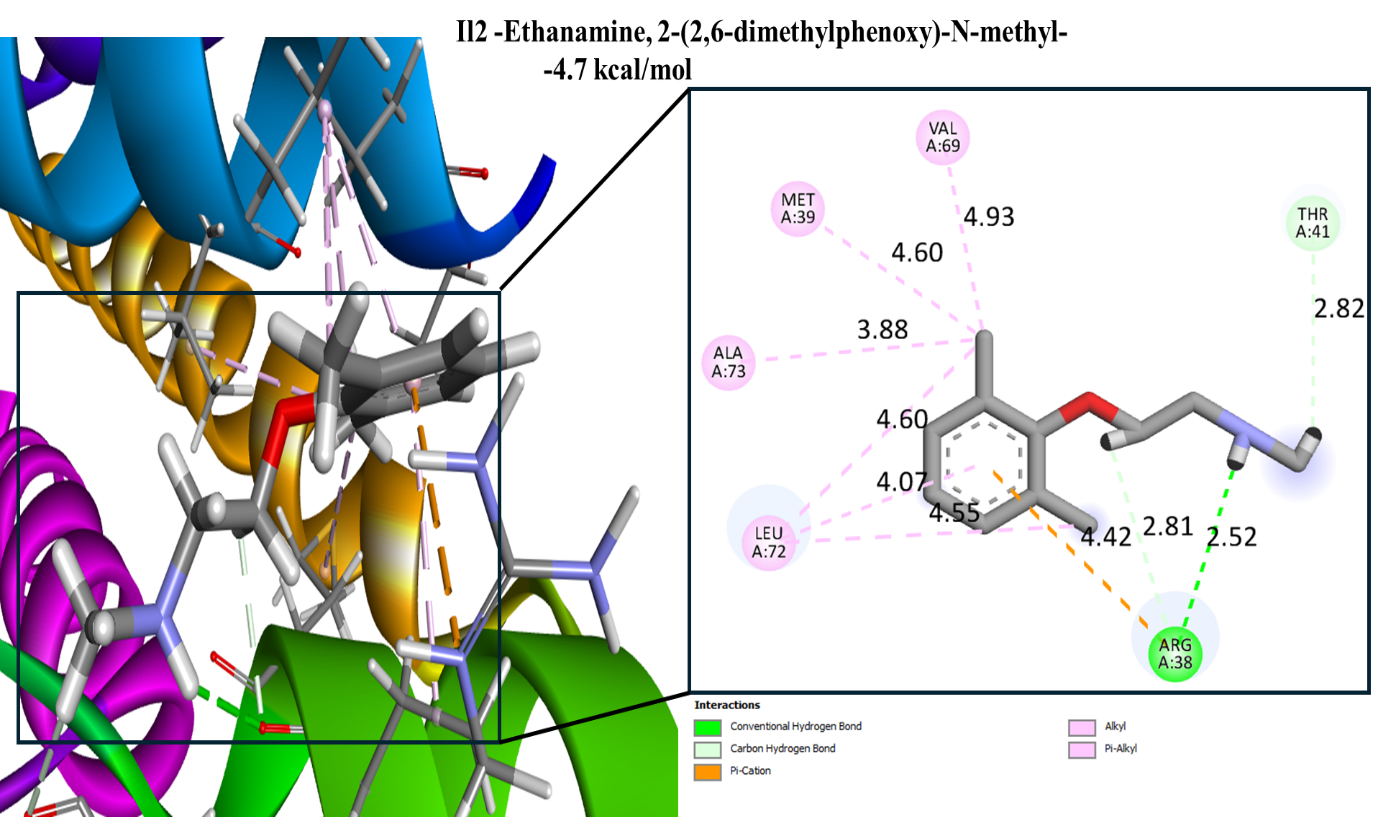 |
| --- |
| 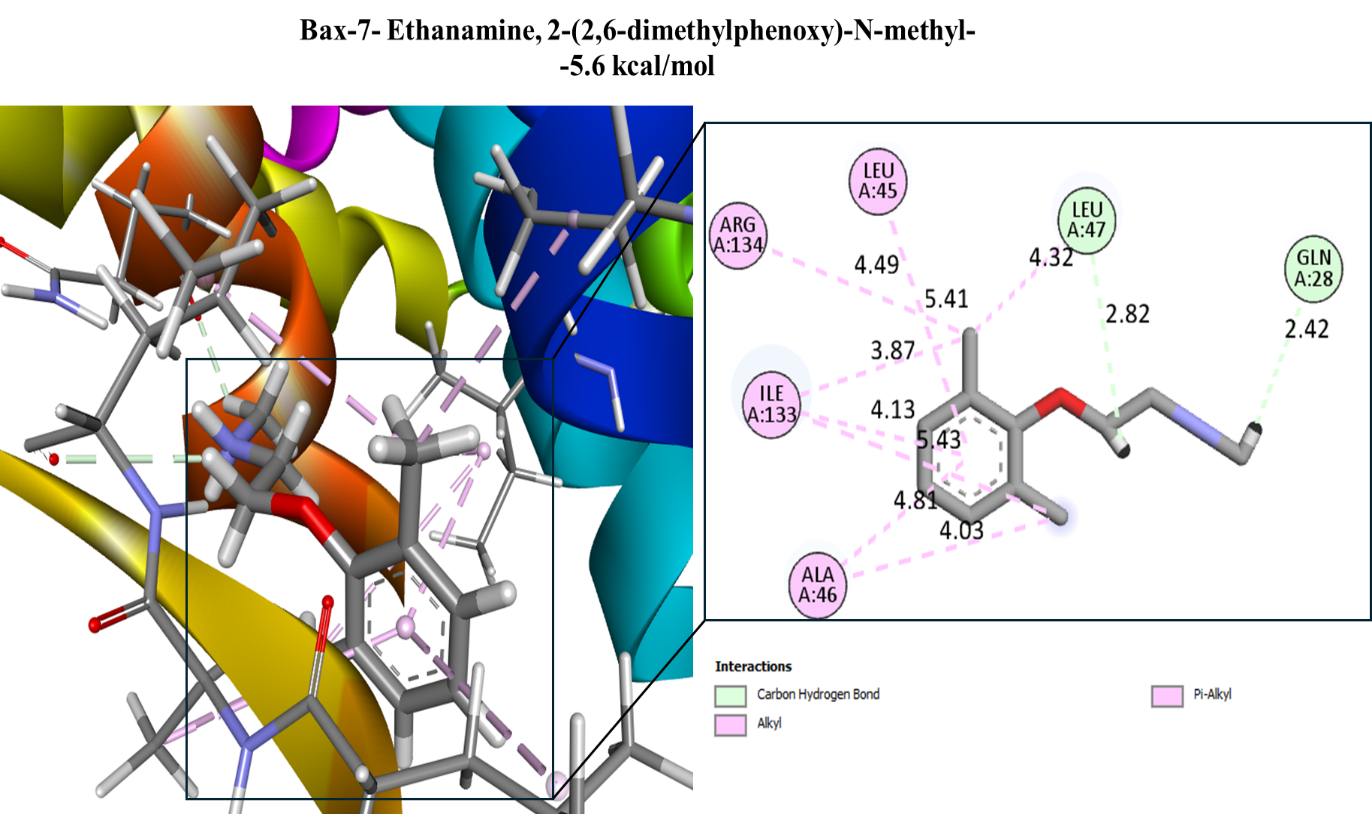 |
| 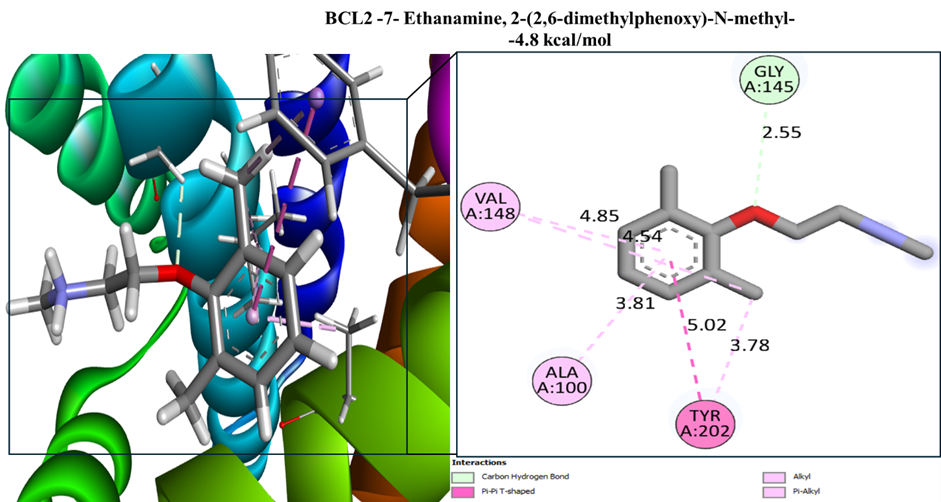 |
| **Supplementary Figure 5:** Docking interactions of Ethanamine, 2-(2,6-dimethylphenoxy)-N-methyl-with active-site residues of target protein receptors |

| 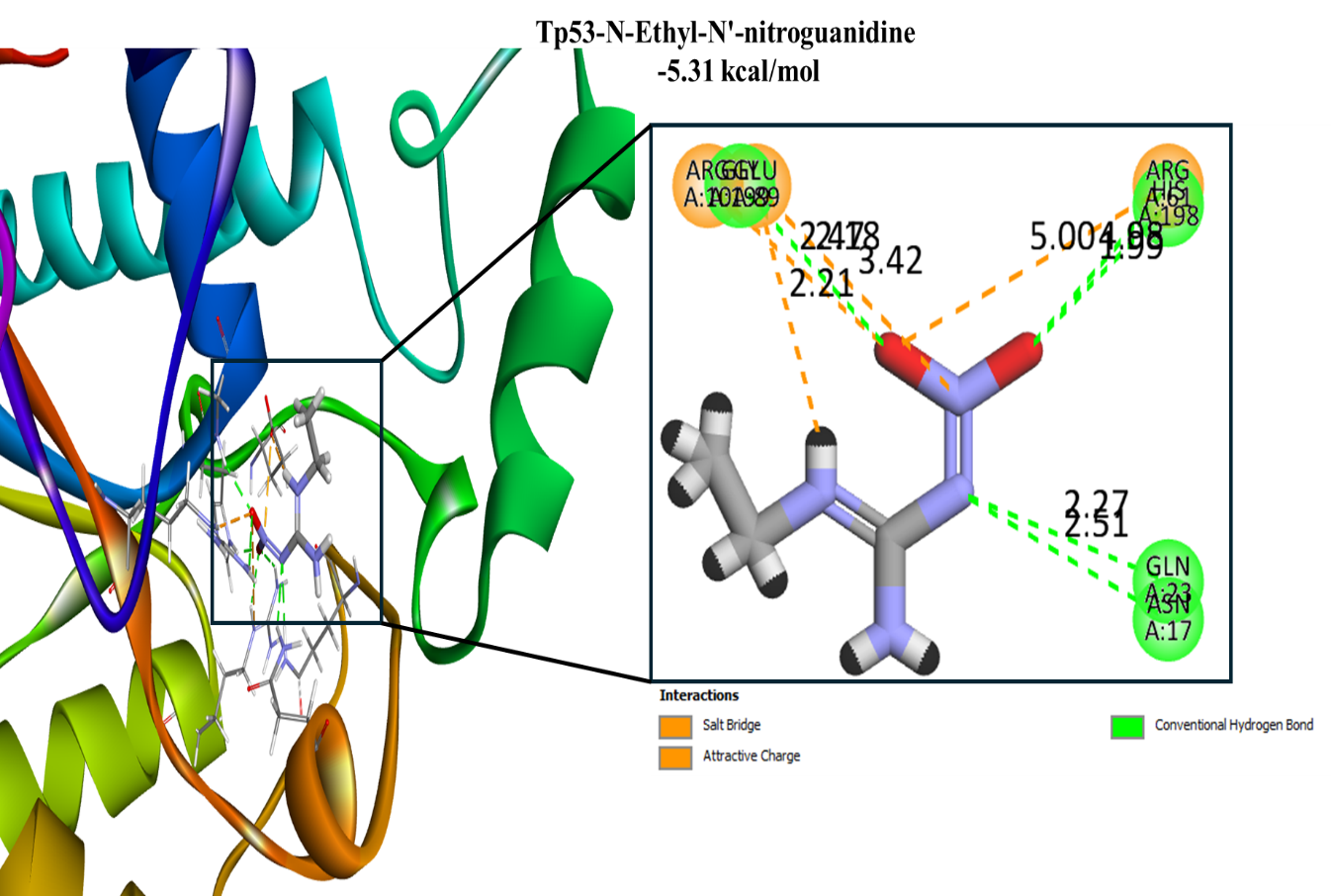 |
| --- |
| 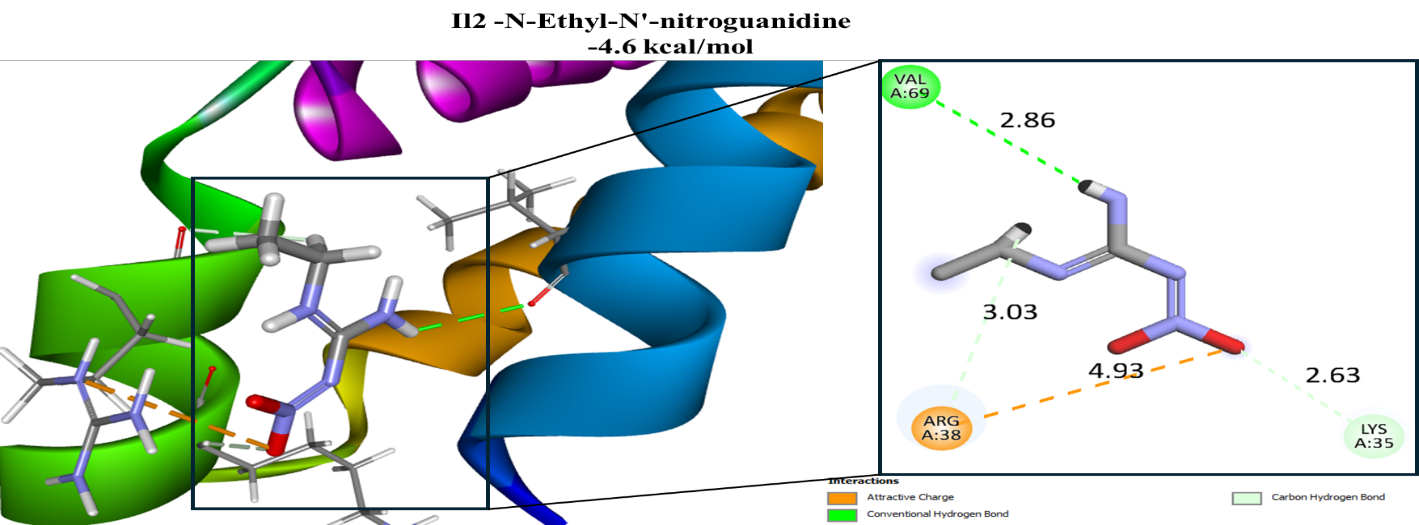 |
| 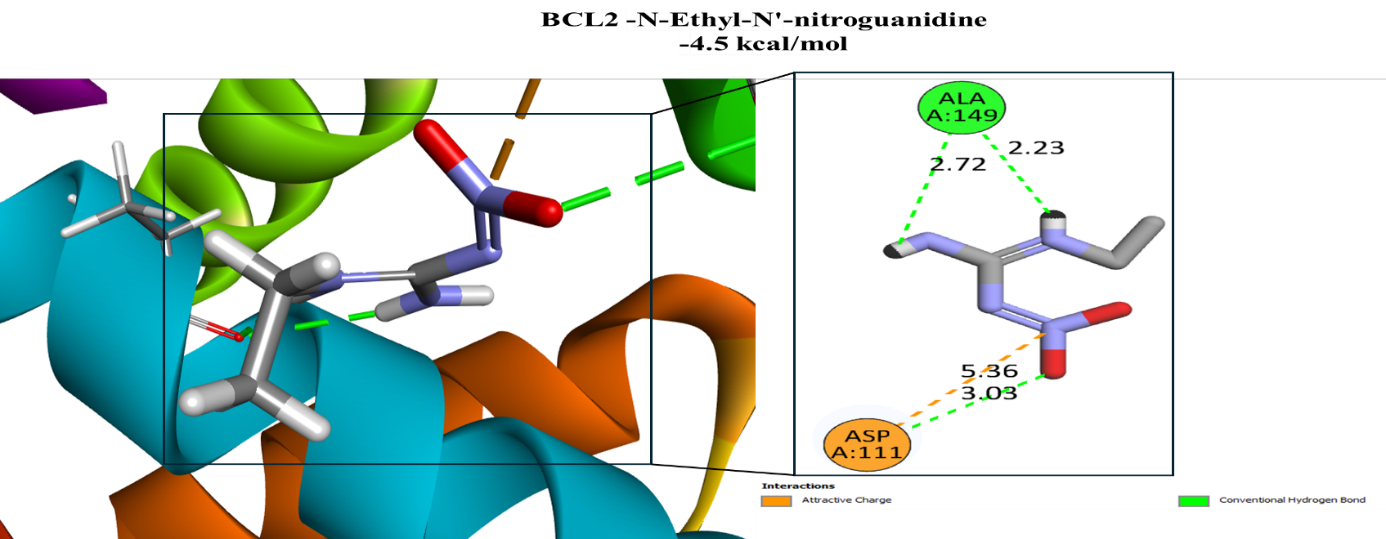 |
| 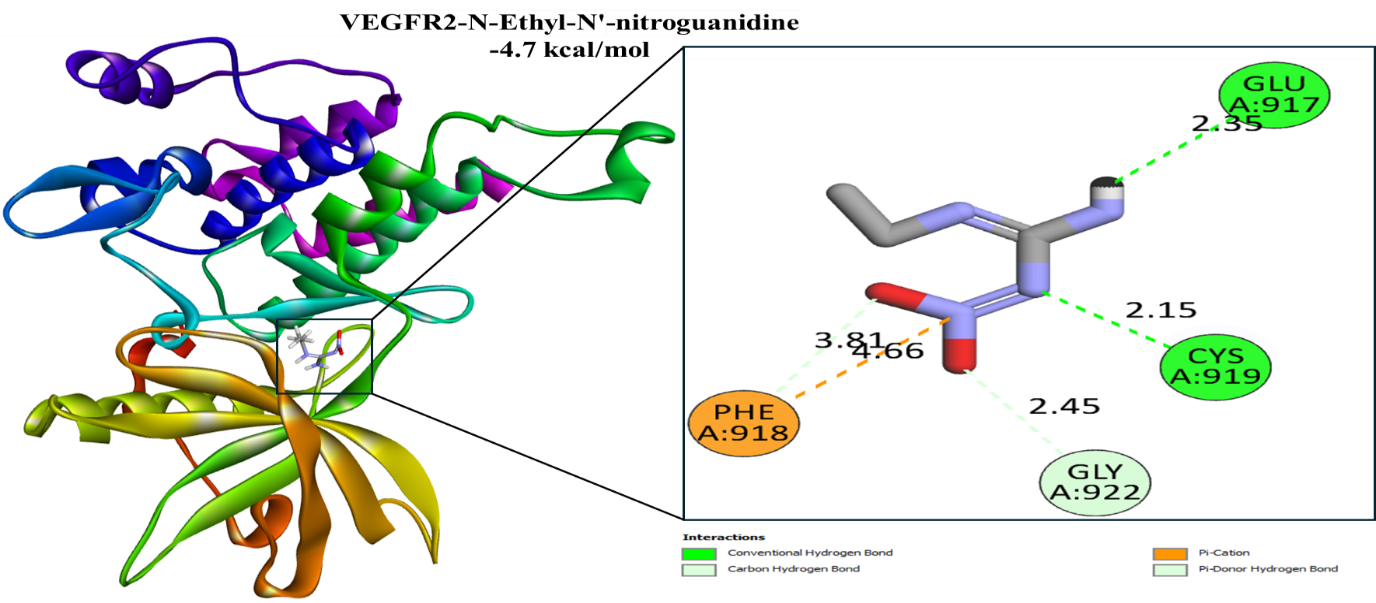 |
| **Supplementary Figure 6:** Docking interactions of N-Ethyl-N'-nitroguanidine with active-site residues of target protein receptors |

| 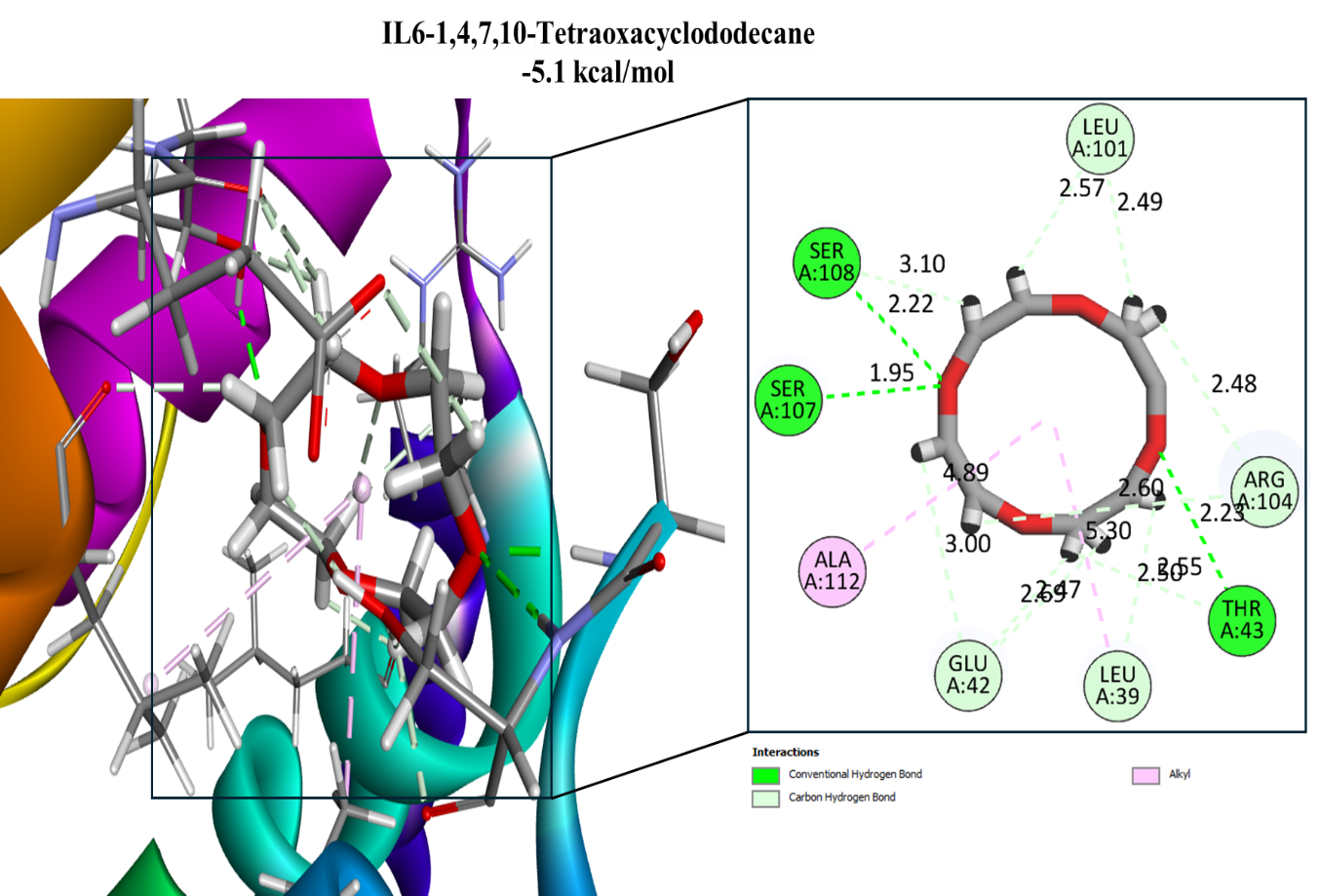 |
| --- |
| 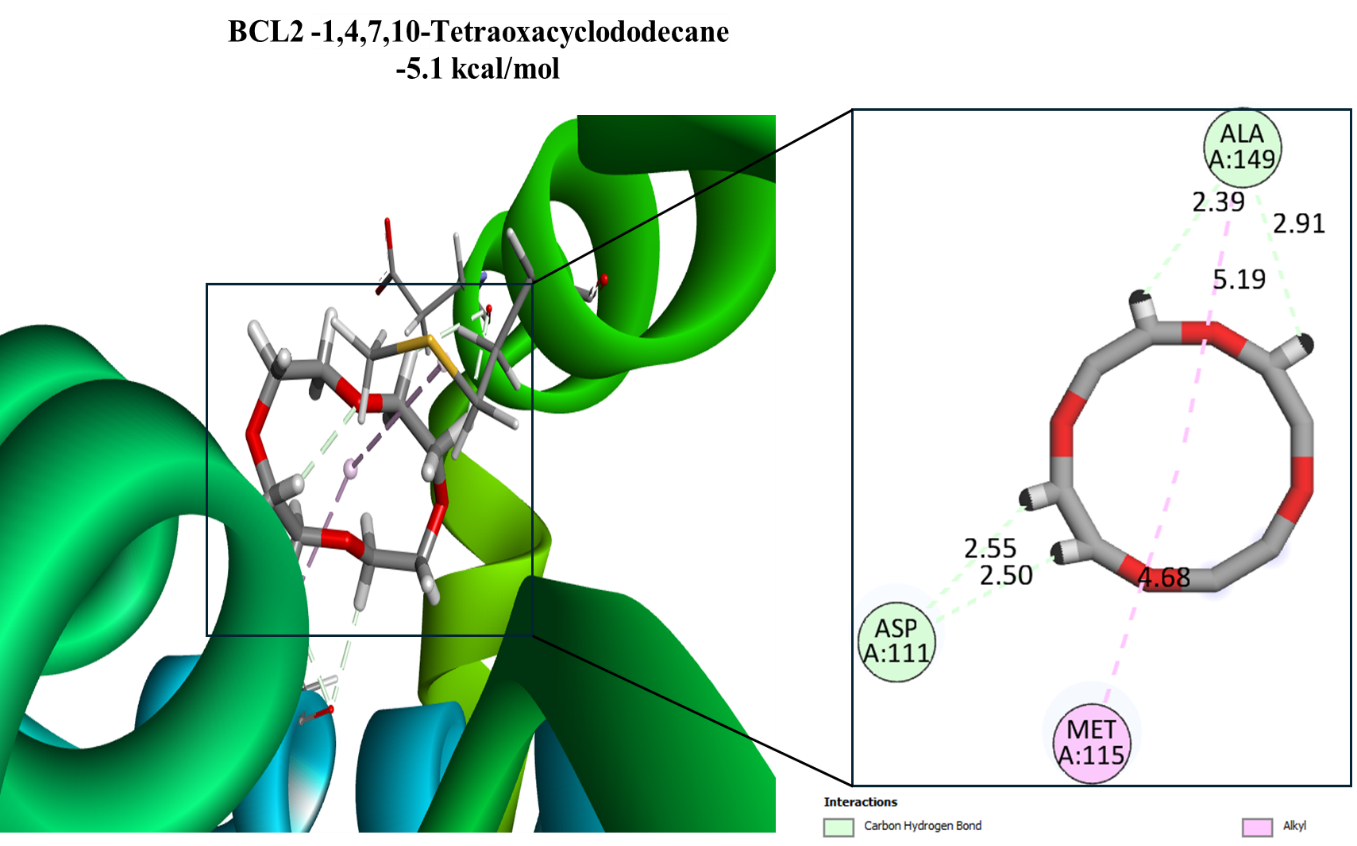 |
| 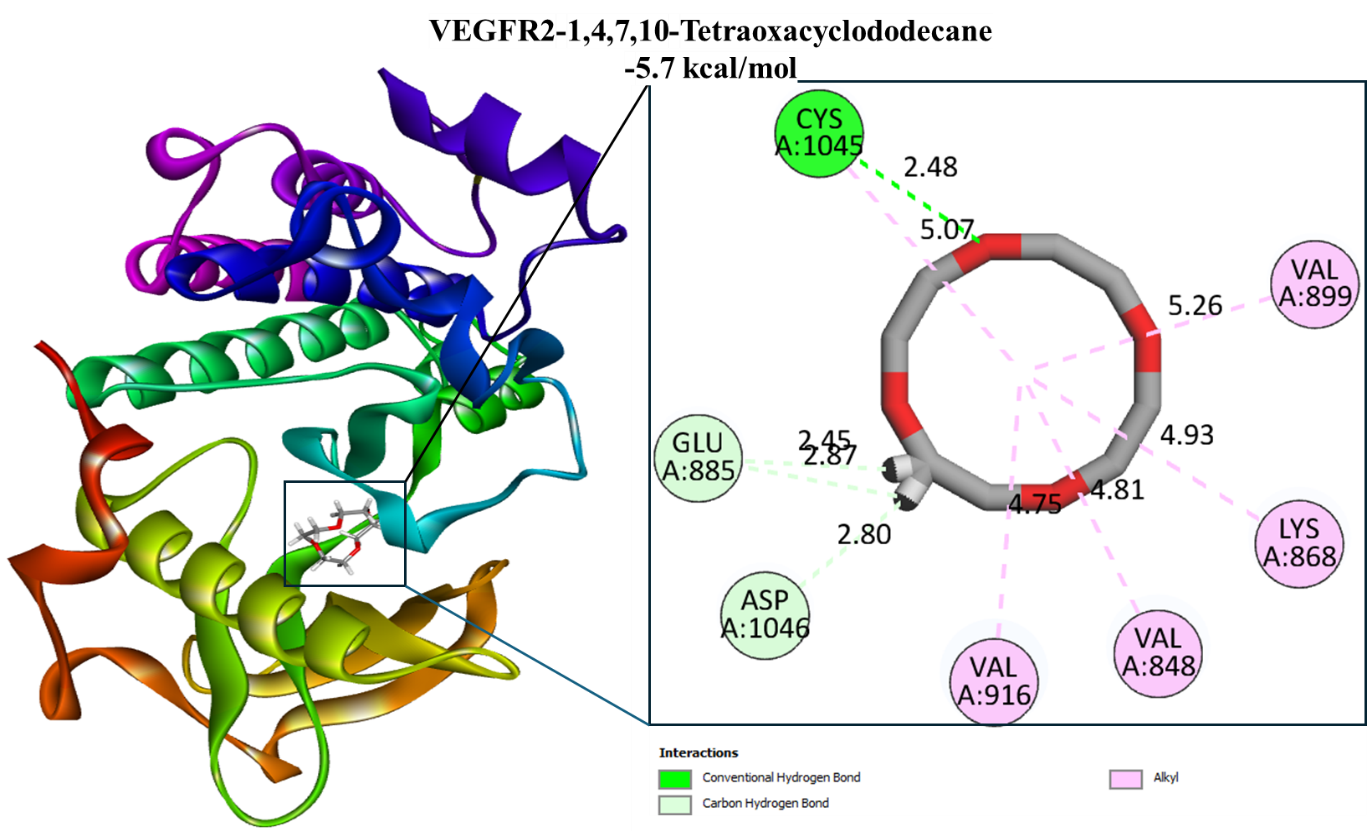 |
| **Supplementary Figure 7:** Docking interactions of 1,4,7,10-Tetraoxacyclododecane with active-site residues of target protein receptors |

| 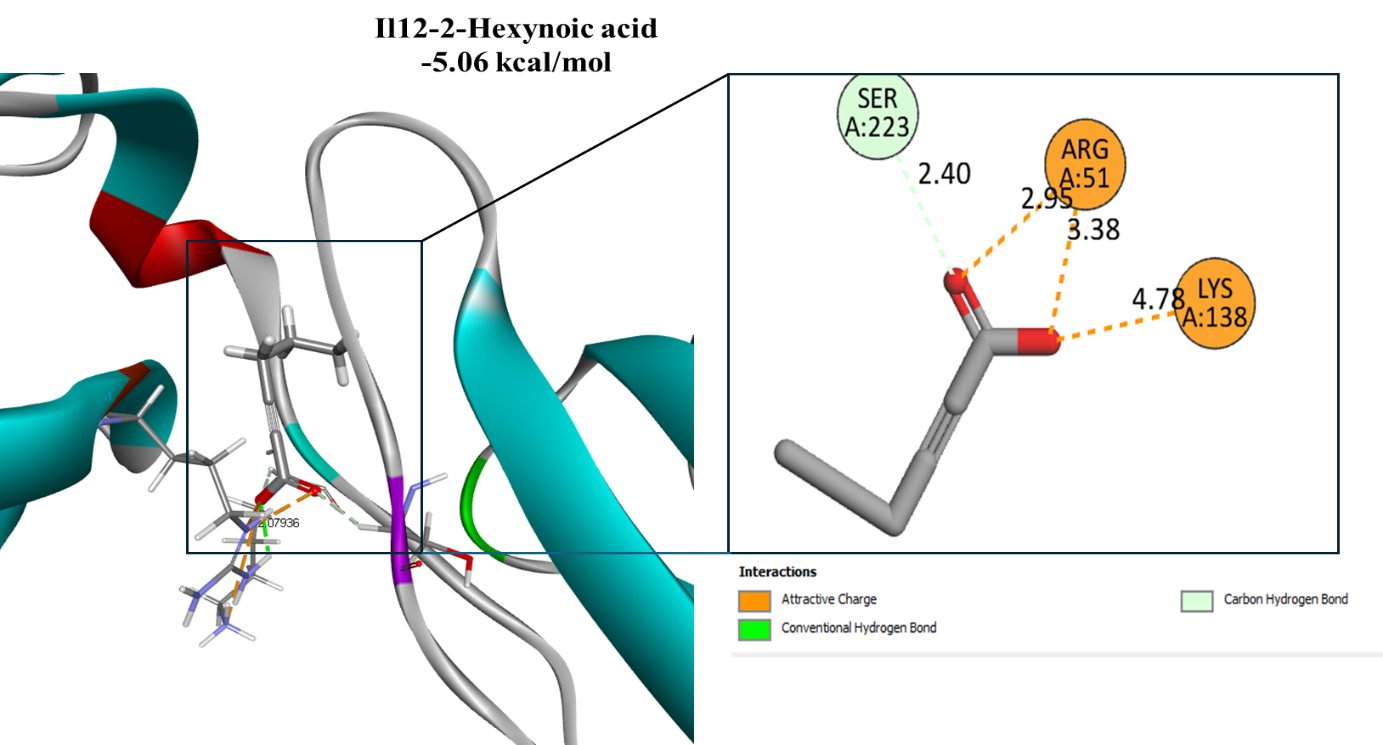 |
| --- |
| 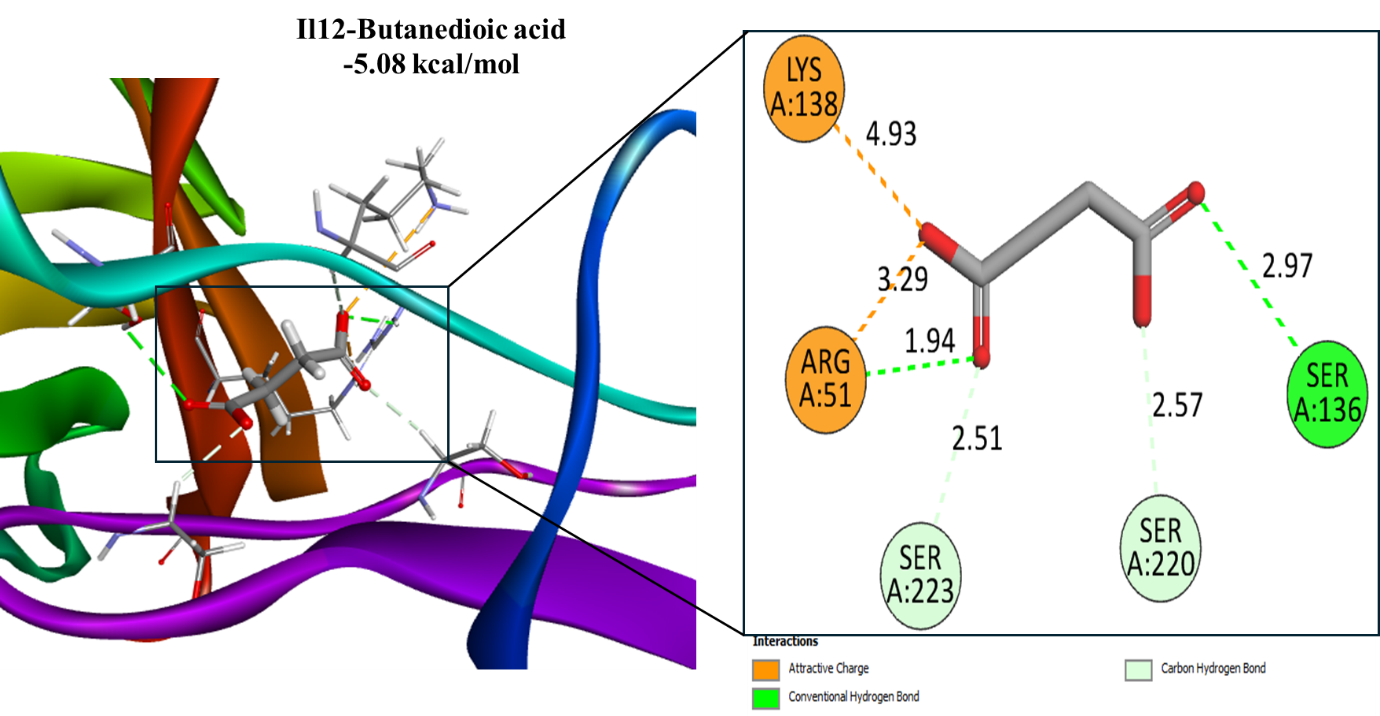 |
| 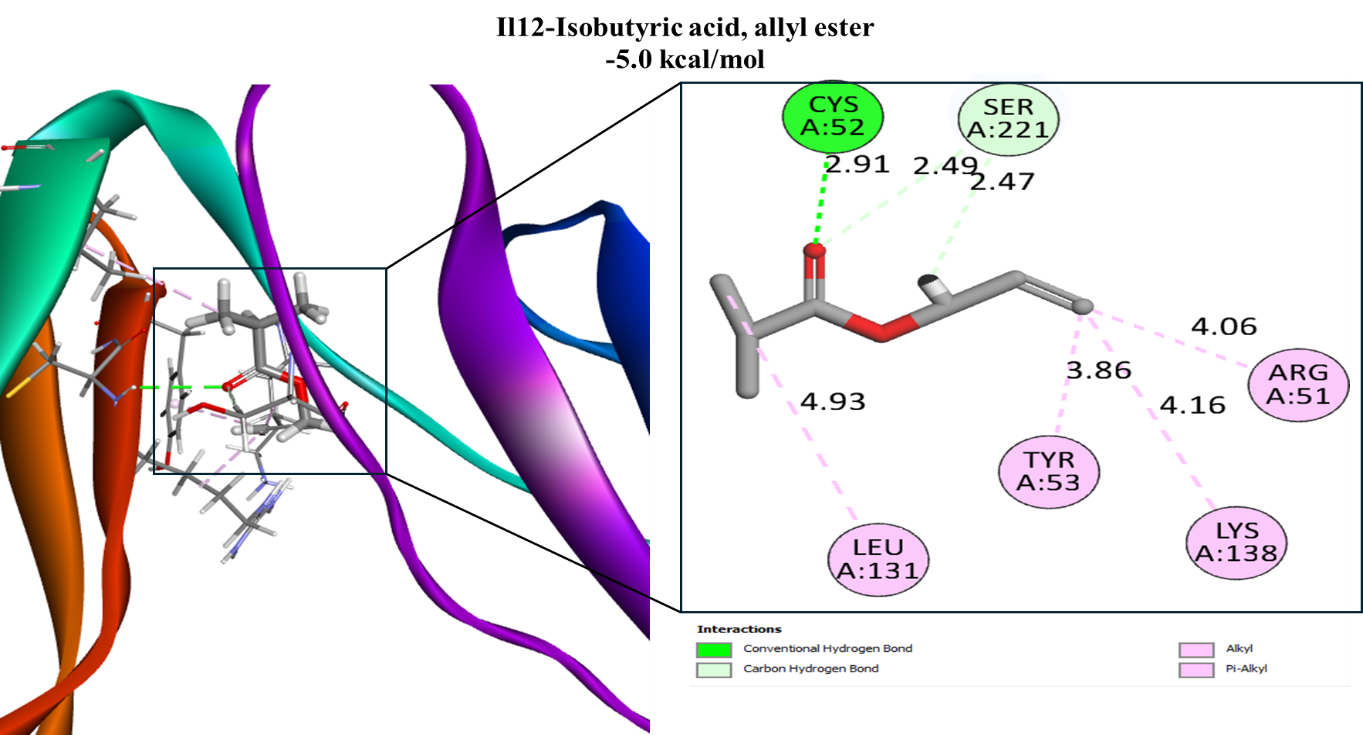 |
| 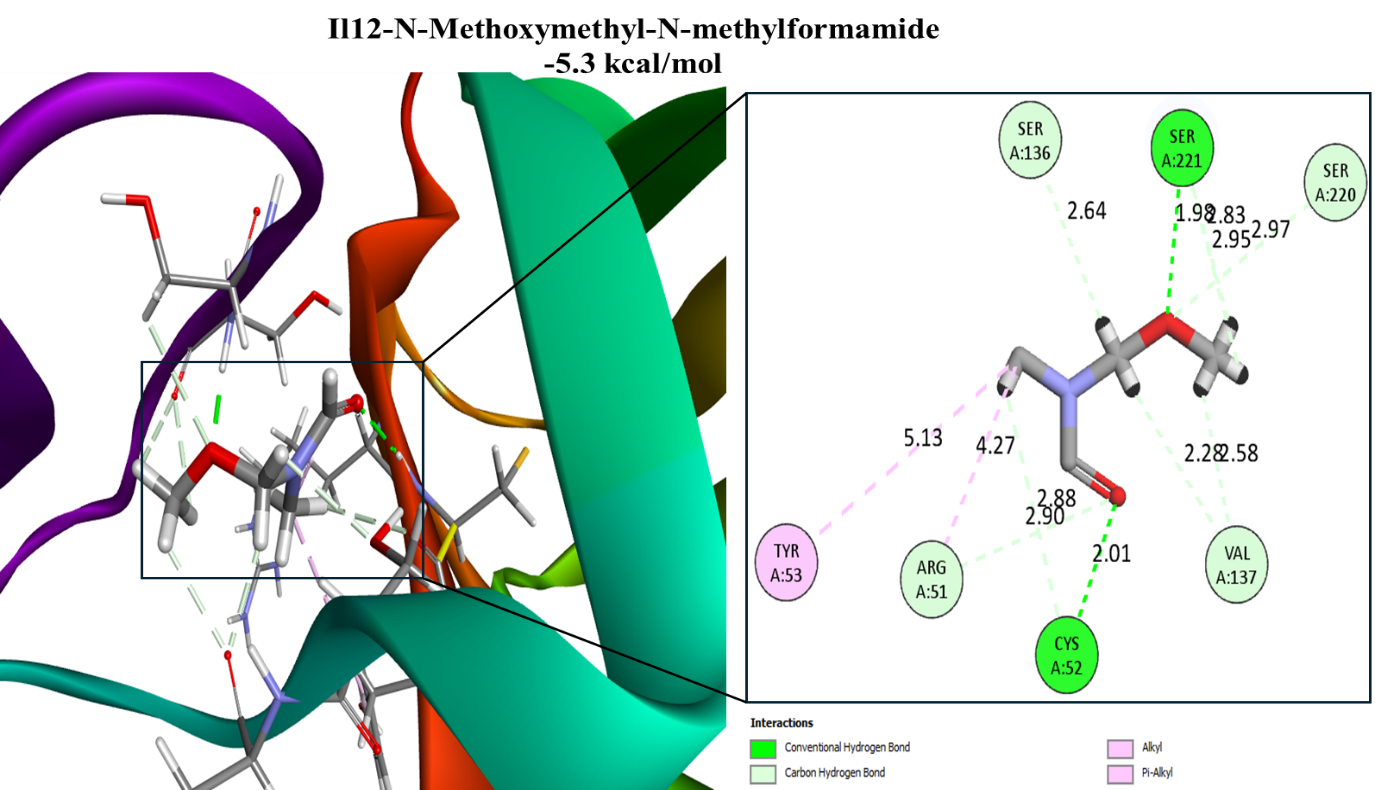 |
| **Supplementary Figure 8:** Docking interactions of il12 with bioactive molecules derived from bee and scorpion venoms |

| 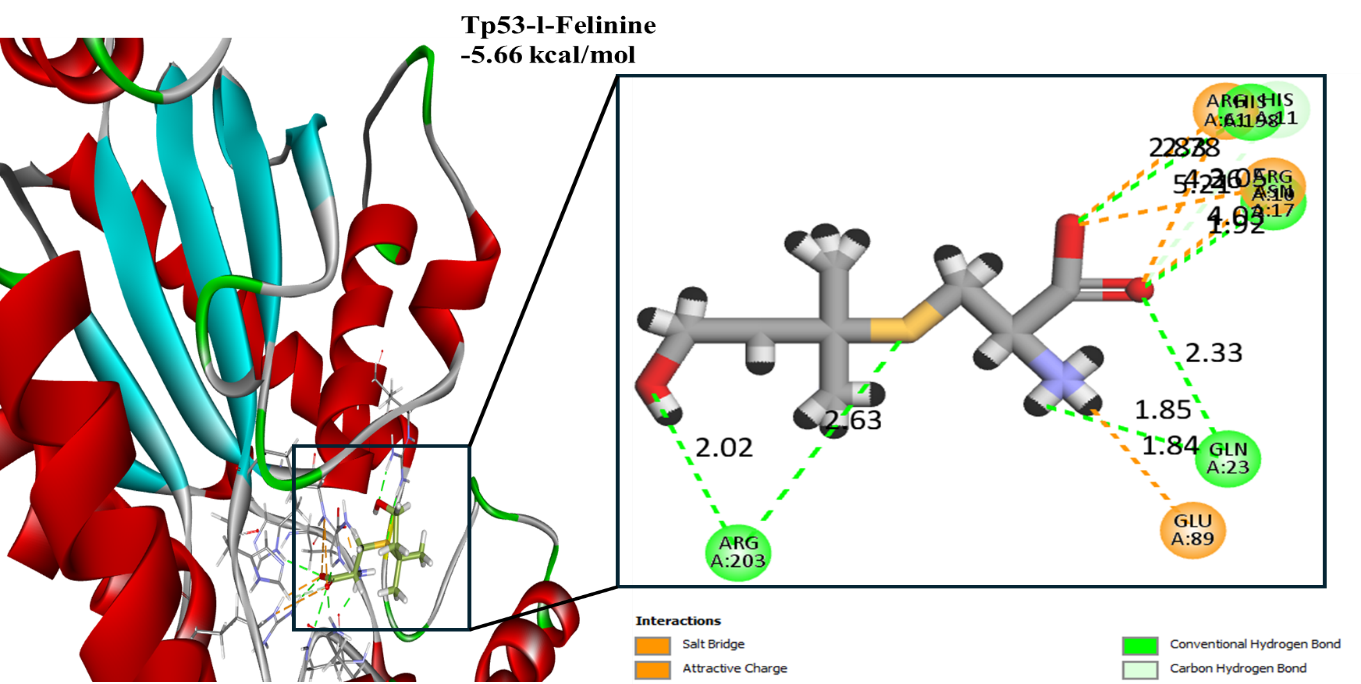 |
| --- |
| 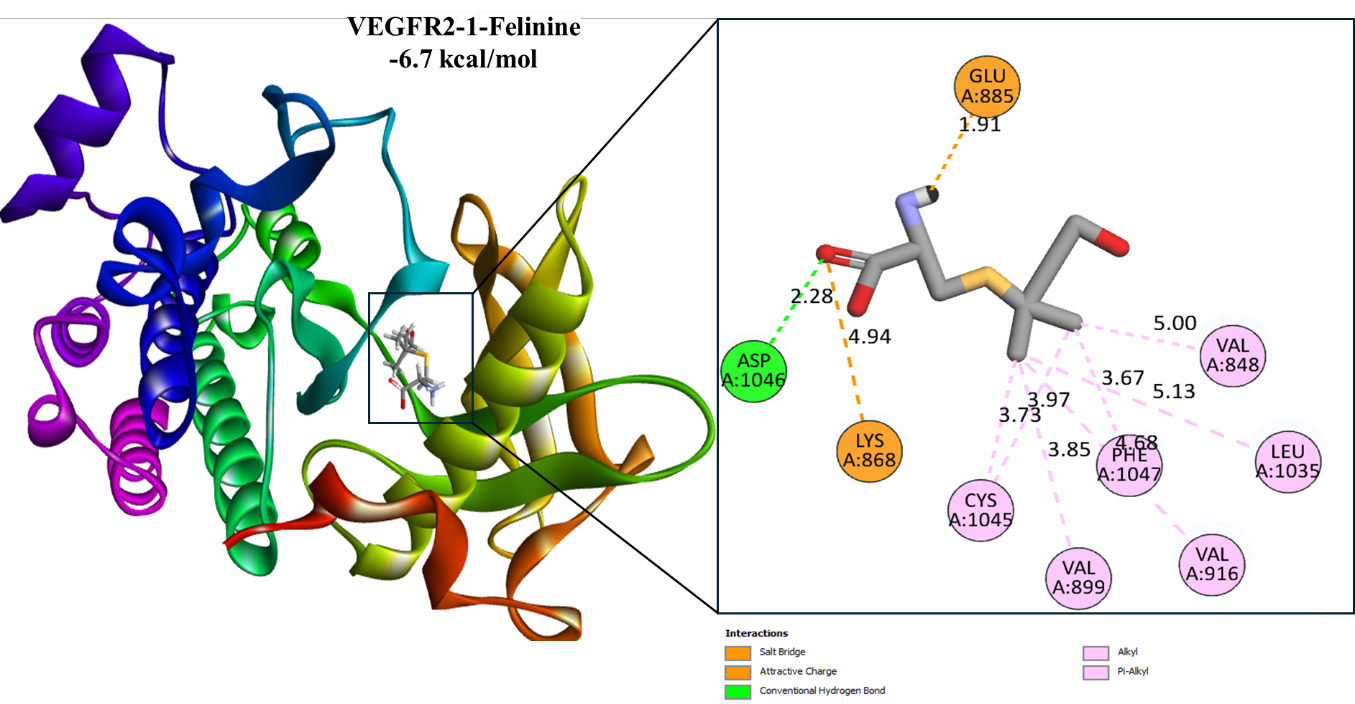 |
| **Supplementary Figure 9:** Docking interactions of l-Felinine with active-site residues of target protein receptors |

| 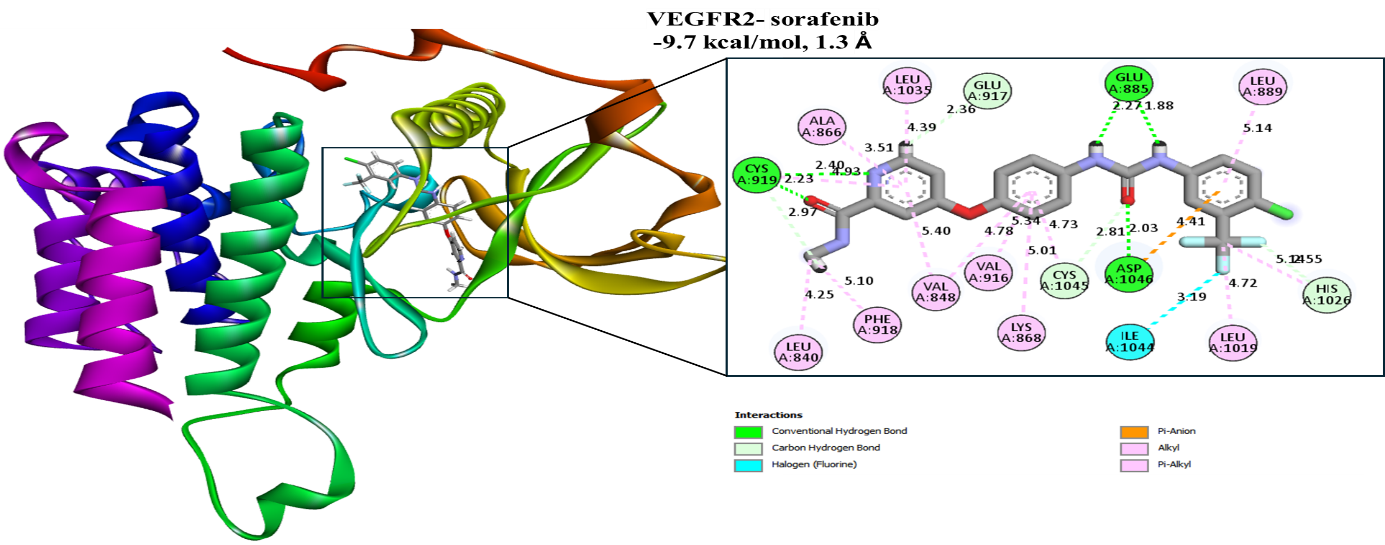 |
| --- |
| **Supplementary Figure 10**: Docking interactions of sorafenib with VEGR2 |
